# Ydj1 governs fungal morphogenesis and stress response, and facilitates mitochondrial protein import via Mas1 and Mas2

**DOI:** 10.15698/mic2017.10.594

**Published:** 2017-10-02

**Authors:** Jinglin L. Xie, Iryna Bohovych, Erin O.Y. Wong, Jean-Philippe Lambert, Anne-Claude Gingras, Oleh Khalimonchuk, Leah E. Cowen, Michelle D. Leach

**Affiliations:** 1 Aberdeen Fungal Group, University of Aberdeen, Institute of Medical Sciences, Foresterhill, Aberdeen, AB25 2ZD, UK.; 2 Department of Molecular Genetics, University of Toronto, Toronto, Ontario, M5S 1A8, Canada.; 3 Department of Biochemistry, University of Nebraska, Lincoln, NE 68588, USA.; 4 Lunenfeld-Tanenbaum Research Institute, Sinai Health System, 600 University Avenue, Toronto, ON, M5G 1X5, Canada.; 5 Nebraska Redox Biology Center, University of Nebraska, Lincoln, NE 68588, USA.; 6 Fred & Pamela Buffett Cancer Center, Omaha, NE 68198, USA.

**Keywords:** Candida albicans, stress, mitochondria, morphogenesis, heat shock, mitochondrial processing peptidases

## Abstract

Mitochondria underpin metabolism, bioenergetics, signalling, development and cell death in eukaryotes. Most of the ~1,000 yeast mitochondrial proteins are encoded in the nucleus and synthesised as precursors in the cytosol, with mitochondrial import facilitated by molecular chaperones. Here, we focus on the Hsp40 chaperone Ydj1 in the fungal pathogen *Candida albicans*, finding that it is localised to both the cytosol and outer mitochondrial membrane, and is required for cellular stress responses and for filamentation, a key virulence trait. Mapping the Ydj1 protein interaction network highlighted connections with co-chaperones and regulators of filamentation. Furthermore, the mitochondrial processing peptidases Mas1 and Mas2 were highly enriched for interaction with Ydj1. Additional analysis demonstrated that loss of *MAS1*, *MAS2* or *YDJ1* perturbs mitochondrial morphology and function. Deletion of *YDJ1 *impairs import of Su9, a protein that is cleaved to a mature form by Mas1 and Mas2. Thus, we highlight a novel role for Ydj1 in cellular morphogenesis, stress responses, and mitochondrial import in the fungal kingdom.

## INTRODUCTION

Mitochondria are essential eukaryotic organelles important for metabolism, bioenergetics, signalling, apoptosis and developmental processes 1. As a consequence, maintenance of mitochondrial activity by preserving protein localization and function is key to survival. Most mitochondrial proteins are synthesised with signal presequences in the cytosol and translocated in an unfolded state through the mitochondrial membranes with the help of chaperone proteins 2,3. In the model yeast *Saccharomyces* *cerevisiae*, the signal presequences are proteolytically removed upon their arrival in the mitochondria by the matrix-located mitochondrial processing peptidases (MPP), encoded by *MAS1* and *MAS2,* enabling final protein folding 4,5. *MAS1* and *MAS2* were originally identified by isolating temperature-sensitive yeast mutants that accumulated uncleaved mitochondrial precursor proteins at the nonpermissive temperature of 37°C 6. This screen also identified *MAS3* and *MAS5*, which correspond to the heat shock transcription factor *HSF1* and the Hsf1 regulated chaperone *YDJ1,* respectively 7,8.

Ydj1 belongs to the Hsp40 class of chaperones, which are homologous to the DnaJ chaperone in *E. coli* and interact with the molecular chaperone Hsp70, accelerating its ATPase activity 9. Deletion of *YDJ1* in *S. cerevisiae* results in slow-growing, stress-sensitive cells, with full Ydj1 function being dependent on farnesylation at the C-terminus 10. The C-terminal domain of Ydj1 binds substrates with a specificity that overlaps with that of Hsp70 11, and together Hsp70 and Ydj1 are capable of refolding denatured luciferase 12. Ydj1 localises to the cytoplasm and endoplasmic reticulum in *S. cerevisiae*, with a small fraction localising to mitochondria 13. It was later discovered that a *ydj1*Δ mutant exhibits defects in mitochondrial import of the nuclear-encoded F_1_β subunit of the mitochondrial ATPase subunit, accumulating greater levels of the precursor form as the temperature is increased from 30°C to 37°C 8,14. Although it is clear that Ydj1 influences mitochondrial protein import, the mechanisms involved remain largely enigmatic.

Much of our knowledge regarding factors governing proteome stability has been derived by studying stress responses in *S. cerevisiae* due to its facile genetics. Dissecting these cellular stress responses is of central importance not only to understand fundamental mechanisms of protein homeostasis, but also to appreciate mechanisms of virulence in fungal pathogens. This is of particular relevance for the commensal fungus* Candida* *albicans, *which has evolved in the mucous membranes and digestive tracts of healthy humans, causing superficial mucosal infections in otherwise healthy individuals upon compromise of host defences 15. Moreover, immunocompromised patients are at risk of developing life-threatening systemic infections with mortality rates of ~40% 16. *C. albicans* has evolved fine-tuned circuitry to sense and respond to diverse stresses relevant to the human host 17. It senses temperature and other host cues, which induce a morphological transition between yeast and filamentous growth, a key virulence trait for dissemination and epithelial invasion 18. Temperature sensing is governed in part by Hsf1 19, which has crucial functions in orchestrating the expression of genes encoding molecular chaperones involved in basal protein homeostasis such as Ydj1, and the heat shock response 20,21. There is a growing appreciation of the mechanisms by which Hsf1 and the molecular chaperone Hsp90 govern *C. albicans* biology 22,23,24,25,26, although the functions of other molecular chaperones regulated by Hsf1 remain a largely uncharted frontier.

The importance of mitochondria in virulence, morphogenesis and stress responses in *C. albicans* has been recently highlighted 27. Mitochondrial proteins have been linked to cell integrity, being required for tolerance to the antifungal drug caspofungin 28,29, and survival during oxidative stress 30. Furthermore, loss of mitochondrial proteins blocks filamentation 31,32, which likely accounts for the attenuated virulence of mutants with defective mitochondria 30. In this study, we investigated the uncharacterised *C. albicans* Hsp40 chaperone Ydj1. We determined that Ydj1 promotes survival in response to oxidative, cell wall and osmotic stress, and is required for growth at high temperature. In addition, we find that Ydj1 is required for filamentation in response to serum and high temperature cues. Utilising a proteomic approach, we identified numerous Ydj1 interactors, discovering a novel role for a pool of mitochondria-associated Ydj1 in facilitating mitochondrial import through the MPP Mas1 and Mas2. We further showed that this unexpected association is important for maintaining mitochondrial morphology and functionality at elevated temperatures.

## RESULTS 

### Ydj1 is required for stress tolerance and morphogenesis in *C. albicans*

**Figure 1:**
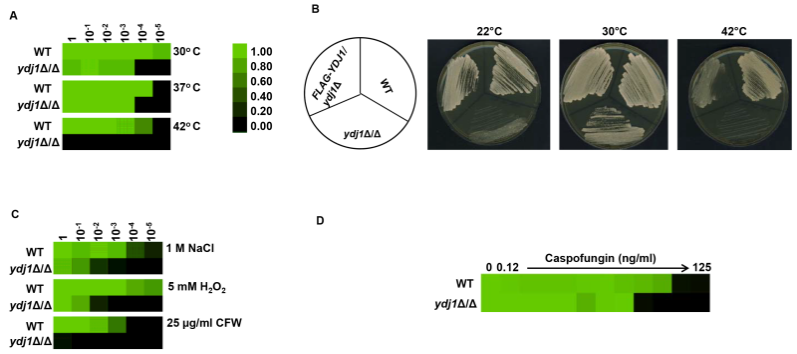
FIGURE 1: Ydj1 is required for stress resistance. **(A)** Ydj1 is required for thermal adaptation. Temperature sensitivity was assayed by monitoring the growth of *C. albicans *wild-type (WT) and *ydj1*Δ/*ydj1*Δ mutant cells at 30°C, 37°C, and 42°C. Cells were diluted 10-fold in YPD medium and grown statically at the indicated temperatures for 72 hours. Growth, measured by absorbance at 600 nm, was averaged for duplicate measurements and normalised relative to the undiluted wild-type strain (see colour bar). **(B)** Fitness and temperature sensitivity of the wild-type strain, the *ydj1*Δ/*ydj1*Δ mutant, and the FLAG-*YDJ1*/*ydj1*Δ complemented strain. Strains were streaked onto YPD plates as diagrammed, and incubated at either 22°C, 30°C or 42°C for 48 hours. **(C)** Ydj1 is important for stress adaptation. Wild-type (WT) and *ydj1*Δ/*ydj1*Δ mutant cells were diluted 10-fold in YPD medium supplemented with 1 M NaCl, 5 mM H_2_O_2_, or 25 µg/ml calcofluor white (CFW), and incubated statically at 30°C for 48 hours. Growth, measured by absorbance at 600 nm, was averaged for duplicate measurements and normalised relative to the undiluted WT strain. **(D)** Ydj1 potentiates caspofungin tolerance. MICs were performed in YPD medium with a caspofungin gradient from 0 to 125 ng/ml, in two-fold dilutions at 30°C. Growth, measured by absorbance at 600 nm, was averaged for duplicate measurements and normalised relative to the undiluted WT strain.

Environmental stress can lead to a breakdown in intracellular protein transport, disrupt the cytoskeleton and trigger global protein unfolding 33, threatening cellular viability. Heat shock proteins are deployed to counteract the detrimental influence of such stressors, preventing protein aggregation and targeting damaged proteins for degradation 34. To establish if Ydj1 is required for responding to stress in *C. albicans*, we first determined if Ydj1 influenced thermal adaptation. Wild-type and *ydj1*Δ/*ydj1*Δ mutant cells were diluted 10-fold in YPD and placed at 30°C, 37°C or 42°C for 72 hours. The *ydj1*Δ/*ydj1*Δ mutant failed to grow at 42°C (Figure 1A), implicating Ydj1 as crucial for growth at elevated temperatures, as is the case in *S. cerevisiae* 8. *S. cerevisiae* Ydj1 is also important for optimal growth at lower temperatures 8, consistent with our observations at 30°C (Figure 1A). To ensure the phenotypes are attributable to deletion of *YDJ1*, the *ydj1*Δ/*ydj1*Δ mutant was complemented with a FLAG-*YDJ1* allele at the native locus, and strains were tested for their ability to grow at 22°C, 30°C or 42°C for 48 hours (Figure 1B). The FLAG-*YDJ1/ydj1*Δ allele partially rescued the slow growth phenotype of the *ydj1*Δ/*ydj1*Δ mutant, validating the mutant phenotypes. Next, we examined the effect of osmotic, oxidative and cell wall stress. The *ydj1*Δ/*ydj1*Δ mutant was sensitive to osmotic and oxidative stress, and was unable to grow in the presence of the cell wall stress agent calcofluor white (Figure 1C). The striking cell wall phenotype prompted us to test whether a *ydj1*Δ/*ydj1*Δ mutant would be hypersensitive to the echinocandin antifungal drug, caspofungin, which exerts a profound cell wall stress in *C. albicans* 35. Indeed, *ydj1*Δ/*ydj1*Δ mutant cells were hypersensitive to caspofungin compared to the wild-type strain (Figure 1D), suggesting that Ydj1 is required for cell wall integrity.

**Figure 2:**
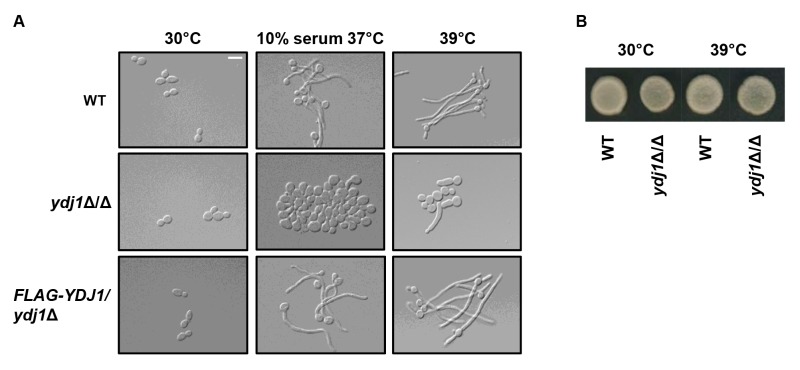
FIGURE 2: Ydj1 is required for adaptation to high temperature. **(A)** Ydj1 regulates temperature-dependent morphogenesis. Wild-type (WT), *ydj1*Δ/*ydj1*Δ mutant and *YDJ1 *complemented strain, FLAG-*YDJ1/ydj1*Δ, were subcultured in YPD medium containing 10% serum and incubated at 37°C for 4 hours or in YPD medium at 39°C for 3 hours; filamentation was compared to cells grown in YPD medium at 30°C. Bar, 10 μm. **(B)** Viability assay after high temperature growth. Viability of the wild-type and *ydj1*Δ/*ydj1*Δ mutant was assayed by spotting 5 μl of culture from cells grown at 30°C or 39°C for 3 hours onto YPD. Plates imaged after 48 hours.

The cell wall of *C. albicans* is critical for the maintenance of cell polarity and interaction with the surrounding environment. Numerous environmental cues induce a morphogenetic switch from a yeast to filamentous form, during which the expression of cell wall proteins is highly regulated 36. Given that the *ydj1*Δ/*ydj1*Δ mutant is hypersensitive to cell wall stressors, and that other heat shock proteins have been implicated in filamentation 37,38, we tested the ability of a *ydj1*Δ/*ydj1*Δ mutant to transition to filamentous growth in response to serum (37°C with 10% serum) or high temperature (39°C) (Figure 2A). The wild type and complemented FLAG-*YDJ1*/*ydj1*Δ strain produced elongated hyphae in response to both conditions tested, whereas the *ydj1*Δ/*ydj1*Δ mutant was blocked in filamentation. Viability of the *ydj1*Δ/*ydj1*Δ mutant was verified after prolonged periods at 39°C by spotting imaged cells onto YPD and incubating at 30°C for 48 hours (Figure 2B). Together, our data demonstrate that Ydj1 is required for optimal growth at 30°C and is essential for high temperature growth. In addition, Ydj1 enables diverse responses to cellular stress and facilitates morphogenesis in response to host-relevant cues.

### Ydj1 interacts with other co-chaperones and the MPP Mas1 and Mas2

**Figure 3:**
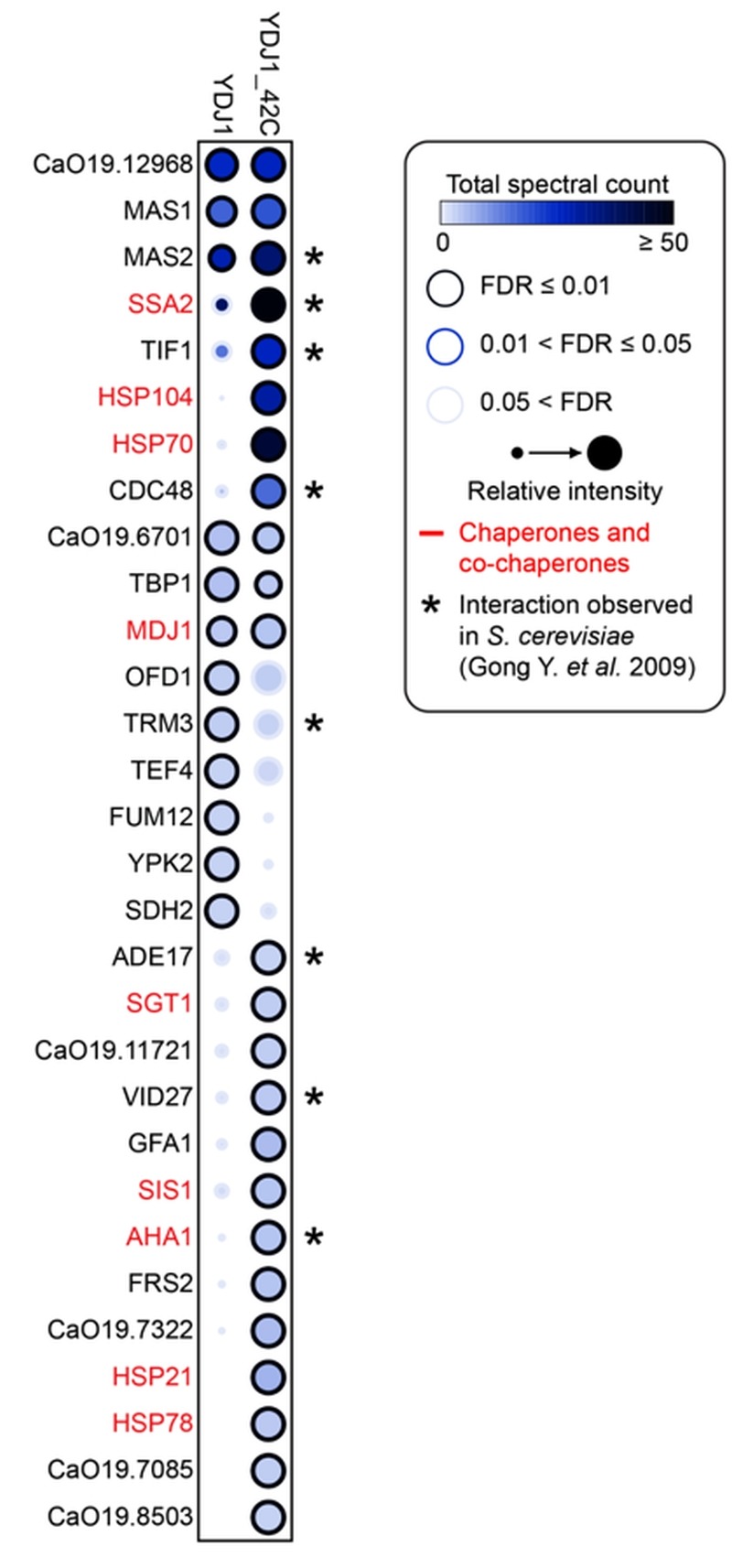
FIGURE 3: The Ydj1 interaction network is modulated by heat shock. AP-MS of 2xFLAG tagged *YDJ1* was performed from FLAG-*YDJ1/ydj1*Δ cells grown at 30°C or subjected to a 15 minute 30°C - 42°C heat shock. Statistically significant Ydj1 interaction partners are shown as a dot plot in which the node color corresponds to the absolute number of spectral count identified for prey proteins, the node size corresponds to the relative abundance between the untreated or heat shock sample, and the node edge represents the SAINT FDR rate at which a given prey protein was observed.

The pleiotropic phenotypes exhibited by the *ydj1*Δ/*ydj1*Δ mutant suggest that it interacts with and aids in the folding of numerous proteins. Recent studies in *S. cerevisiae* identified 64 physical interactions with Ydj1, including 11 chaperone interactors 39,40. To identify Ydj1 interactors in *C. albicans* that might influence stress responses, such as heat shock, and morphogenesis, we performed co-immunoprecipitation coupled to mass spectrometry. Wild-type and 2xFLAG-*YDJ1*/*ydj1*Δ strains were grown at 30°C or subjected to a 30°C - 42°C heat shock. We identified 30 proteins that interact with Ydj1 in the absence or presence of heat shock, including eight chaperone proteins (Figure 3). The interaction of Ydj1 with additional chaperone proteins was expected based on findings in *S. cerevisiae*, where Ydj1 interacts with Hsp104 41, Sis1 and Hsp78 39, working with Hsp104 and the Hsp70 co-chaperone Ssa1 to support protein refolding 41. We identified Hsp104, Hsp70, the Hsp70 co-chaperone Ssa2, Sis1, and Hsp78 as interactors of Ydj1 in* C. albicans*, suggesting that it has a conserved role in protein folding. In addition to these chaperones, we also found Ydj1 to interact with the mitochondrial chaperone Mdj1, the Hsp90 co-chaperone Aha1, and Hsp21 (Figure 3).

**Figure 4:**
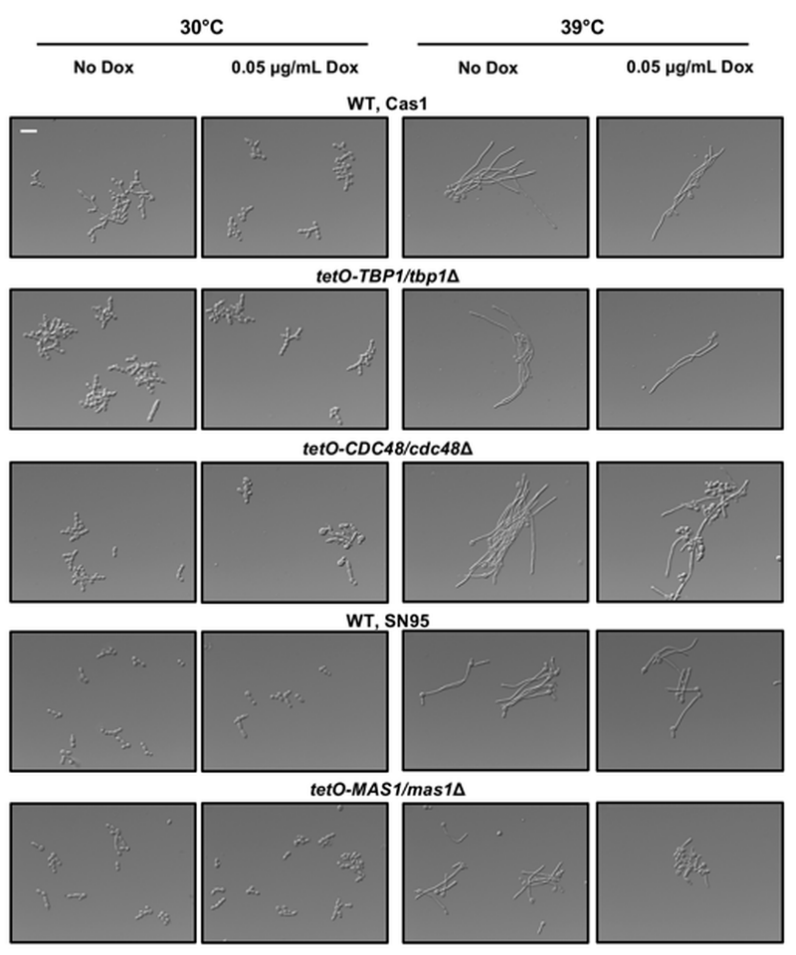
FIGURE 4: Mas1 and Cdc48 contribute to filamentation in response to elevated temperature. The indicated doxycycline-repressible conditional expression strains and their respective wild-type counterparts were cultured in the absence or presence of 0.05 μg/mL doxycycline (Dox) in YPD at 30°C for 16 hours. Cells were subcultured under the same conditions and grown at 30°C or 39°C for 3 hours before imaging. Scale bar represents 20 μm.

Our observations that the *ydj1*Δ/*ydj1*Δ mutant is defective in filamentation suggests that Ydj1 is required for the initiation of filamentous growth. Hsp21 contributes to the formation of filaments 38, but homozygous deletion mutants are not fully blocked in the yeast to filament transition implicating additional factors. Analysis of our Ydj1 interactors highlighted several positive regulators of filamentation in response to serum: Gfa1 42, Cdc48, Tbp1 and Mas1 43. However, these proteins are essential for *C. albicans* growth, thus it is important to distinguish specific functions in filamentation from confounding effects on viability. We tested the *tetO-CDC48/cdc48*Δ and *tetO-TBP1/tbp1*Δ doxycycline-repressible strains from the GRACE (gene replacement and conditional expression) collection 44, along with a *tetO-MAS1/mas1*Δ strain that we constructed, in conditions that supported viability for morphogenesis in response to elevated temperature of 39°C (Figure 4). In the presence of 0.05 μg/mL doxycycline to repress target gene expression, the *tetO-TBP1/tbp1*Δ strain filamented robustly, whereas the *tetO-CDC48/cdc48*Δ and *tetO-MAS1/mas1*Δ strains displayed a partial defect, with a mixture of yeast and filamentous cells present. The partial defect could be due to stochastic differences in the level of target protein still present in the cell at the point of depletion. Given that both Mas1 and Cdc48 are involved in mitochondrial function in *S. cerevisiae* 5,45, and that mitochondria play a role in filamentation 32, our data suggests that Yjd1 may promote filamentation in part via proteins important for mitochondrial function.

### Mas1 and Mas2 are required for mitochondrial function in *C. albicans*

**Figure 5:**
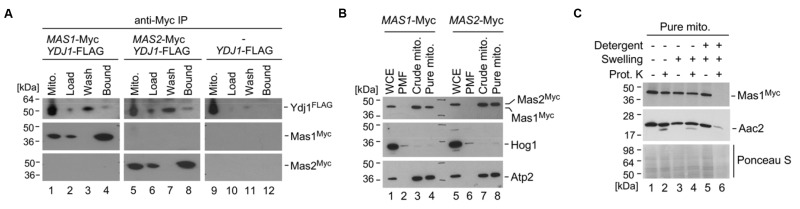
FIGURE 5: Ydj1 interacts with Mas1 and Mas2, which are localised to the mitochondria. **(A) **Gradient purified mitochondria from 2×FLAG-*YDJ1/ydj1*, 2×FLAG-*YDJ1/ydj1 MAS1*-Myc and 2×FLAG-*YDJ1/ydj1 MAS2*-Myc incubated for 6 hours at 37^o^C were lysed and used for the co-immunoprecipitation assay. A physical interaction between Ydj1 and Mas1 or Mas2 was determined by positive anti-FLAG and anti-Myc (Santa Cruz Biotechnology) antibody staining in the Bound fractions. **(B)** Whole-cell extracts (WCE), post-mitochondrial fractions (PMFs), crude and gradient-purified (pure) mitochondria were obtained from yeast cultures grown in YPD at 30^o^C for 16 hours. Fractions were analysed by SDS-PAGE and immunoblotting with antibodies against the Myc epitope (Roche) used to tag Mas1 and Mas2. The purity of the mitochondrial fractions was determined by the absence of a Hog1-specific band normally present in the cytosolic fractions. Antibodies against the inner mitochondrial protein Atp2 were used as a marker for the mitochondrial fraction. **(C)** Mas1 is localised to the mitochondrial matrix. Gradient-purified mitochondria harboring Myc-tagged Mas1 were subjected to osmotic disruption of the outer mitochondrial membrane (Swelling) and lysis with dodecyl maltoside (Detergent) to disrupt the inner membrane in combination with Proteinase K (Prot. K) treatment. The fractions were subjected to Western blot analysis using anti-Myc antibodies to visualise Mas1 localisation, and anti-serum against the inner mitochondrial membrane protein Aac2 used as a marker for submitochondrial localization. Ponceau S staining was used to ascertain the loading of the mitochondrial fractions.

Our most striking interaction detected was with the MPP enzymes Mas1 and Mas2 (Figure 3). They remain uncharacterised in *C. albicans*, but the orthologues in *S. cerevisiae* are required for cleaving the N-terminal targeting signal off nuclear encoded mitochondrial proteins upon import 6. *S. cerevisiae* cells depleted of one or both MAS subunits continue to import precursor proteins in the mitochondria, but fail to cleave them, leading to cell death 46. The physical interaction identified between Ydj1 and Mas1/Mas2 by mass spectrometry suggests shared functional relationships. To validate the physical interaction, and ensure that the MPPs did not adventitiously bind to Ydj1 after cell disruption, we performed co-immunoprecipitation from gradient purified mitochondria coupled to Western blot analysis. Immunoprecipitation of Myc-tagged Mas1 or Mas2 with anti-Myc resin co-purified both the Myc-tagged Mas proteins and FLAG-tagged Ydj1 (Figure 5A). For the control strain lacking the tagged *MAS* alleles, Ydj1 was present in the input (mitochondria) but was not immunoprecipitated (bound fraction) (Figure 5A).

To determine if Mas1 and Mas2 are important for mitochondrial function in *C. albicans*, we first looked at Mas1 and Mas2 localisation. Strains with the C-terminally Myc-tagged Mas1 and Mas2 proteins were grown in YPD for 16 hours, after which whole-cell extracts, post-mitochondrial fractions, crude and gradient purified (pure) mitochondria were extracted and analysed by Western blotting. Bands corresponding to Mas1 and Mas2 were detected in the whole cell extracts, crude and pure mitochondrial extracts (Figure 5B). The inner mitochondrial protein, Atp2, served as a positive control, being detected in the same samples (Figure 5B). The purity of the mitochondrial fractions was determined by probing for the cytosolic protein Hog1, which was abundant in the whole cell extracts and post-mitochondrial (PMF) fractions, but is extremely scarce in the enriched mitochondrial fractions (Figure 5B). Lastly, we performed an assay to determine where Mas1 is localised in the mitochondria. The organelles were purified from the cells expressing Mas1-Myc and subjected to a series of treatments with or without addition of the exogenous proteinase K. Similar to Aac2, an inner mitochondrial membrane protein, Mas1-Myc remained inaccessible to proteinase K (PK) both in intact and osmotically disrupted (Swelling) organelles. However, both proteins were degraded when osmotically challenged mitoplasts were incubated with PK in the presence of dodecyl maltoside (Detergent), which disrupts the inner mitochondrial membrane. These data strongly suggest that Mas1 is a mitochondrial matrix protein (Figure 5C). Similar results were obtained for the C-terminally Myc-tagged Mas2 (data not shown).

**Figure 6:**
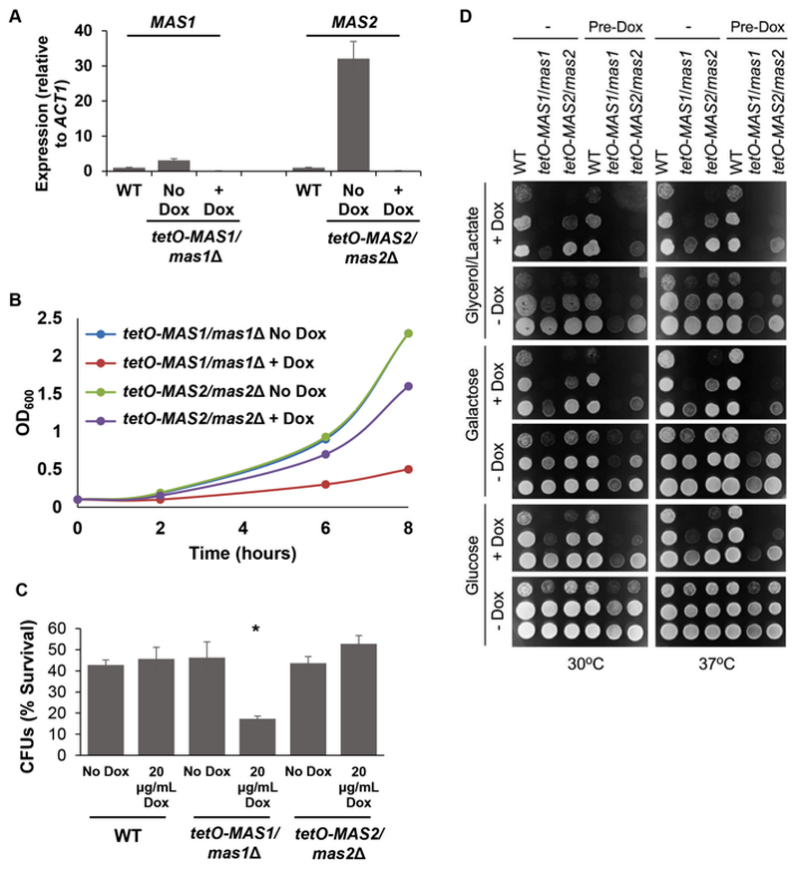
FIGURE 6: The MPP Mas1 and Mas2 are required for mitochondrial function. **(A)** Assessment of the conditional mutants for *MAS1*and *MAS2*. *MAS1* and *MAS2* are depleted using the tetracycline repressible promoter. *C. albicans* wild-type (WT), *tetO-MAS1/mas1*Δ and *tetO-MAS2/mas2*Δ cells were grown in the absence (No Dox) or presence (+ Dox) of 20 µg/ml doxycycline for 24 hours and then subcultured in the same conditions for 6 hours; transcript levels of *MAS1* and *MAS2* were measured and normalised to the *ACT1* loading control. **(B)** Depletion of *MAS1* or *MAS2* reduces *C. albicans* growth. The *tetO-MAS1/mas1*Δ and *tetO-MAS2/mas2*Δ strains were grown in the absence (No Dox) or presence (+ Dox) of 20 µg/ml doxycycline for 24 hours, before being subcultured under the same conditions. Growth was measured for a further 8 hours and OD_600_ was plotted against strains in the absence of depletion. **(C)** Mas1 is required for oxidative stress survival. The impact of oxidative stress on the *tetO-MAS1/mas1*Δ and *tetO-MAS2/mas2*Δ strains and the respective wild type was determined by measurement of CFUs after a 1 hour stress with 5 mM H_2_O_2_. Percent CFUs was determined relative to untreated cells. Students paired, two-tailed t-test, * p < 0.05. **(D)** Depletion of either Mas1 or Mas2 leads to respiratory growth defects. The WT (SN95), *tetO-MAS1/mas1* and *tetO-MAS2/mas2* strains were grown in the absence (-) or presence (pre-dox) of 20 μg/ml doxycycline in YPD for 24 hours. The cells were then serially diluted (with the lowest dilution of A_600_=1) and plated on YP-based plates containing various carbon sources with (+ Dox) or without (- Dox) doxycycline and incubated at 30^o^C or 37^o^C for 36 hours.

As Mas1 and Mas2 are essential proteins in *S. cerevisiae* 46, we generated conditional mutants to assess mitochondrial morphology and function. First, *MAS1* and *MAS2* were placed under the *tetO *promoter and gene expression was assessed. Growth in the presence of doxycycline significantly reduced expression of *MAS1* and *MAS2 *in the *tetO-MAS1/mas1*Δ and *tetO-MAS2/mas2*Δ strains compared to wild-type cells (Figure 6A). Second, viability was assessed, whereby we observed that both strains exhibited reduced growth (Figure 6B), but remained viable during phenotypic analysis. Therefore, the *tetO* promoter sufficiently reduced expression of *MAS1* and *MAS2*, however, it has been previously reported that doxycycline affects mitochondrial function 47. As such, to ensure that results were not affected due to the addition of doxycycline, all experiments also included a doxycycline only control.

Defects in mitochondrial function cause hypersensitivity to oxidative stress and impaired capacity to grow on non-fermentable carbon sources in *S. cerevisiae* 48. To determine if depletion of *MAS1 *or *MAS2* also confers hypersensitivity to oxidative stress, as was the case with deletion of *YDJ1* (Figure 1C), we monitored survival of our *tetO-MAS1/mas1*Δ and *tetO-MAS2/mas2*Δ strains in response to oxidative stress induced by treatment with 5 mM H_2_O_2_ for one hour after depletion of target gene expression. We observed a significant decrease (p < 0.05) in cell survival in response to oxidative stress upon depletion of *MAS1*, but not *MAS2 *(Figure 6C), likely due to a more auxiliary role of the latter protein. Growth of our *tetO-MAS1/mas1*Δ and *tetO-MAS2/mas2*Δ strains was also measured on fermentable medium, YPD (contains 2% glucose as a sole carbon source), fermentable medium that requires mitochondrial function, YP-galactose (2%), and non-fermentable YP-Glycerol/Lactate (each at 2%). Strains were grown in YPD or YPD containing 20 μg/ml doxycycline for 24 hours before being spotted on YP-based plates with or without 20 μg/ml doxycycline containing glucose, galactose or glycerol/lactate (Figure 6D). Plates were incubated at 30°C or 37°C. The wild-type strain grew well on all carbon sources (Figure 6D). However, both the *tetO-MAS1/mas1*Δ and *tetO-MAS2/mas2*Δ strains exhibited growth defects on YP-galactose and YP-glycerol/lactate in the presence of doxycycline (Figure 6D). Strikingly, the *tetO-MAS1/mas1*Δ strain pre-grown in doxycycline and plated on YP-galactose or YP-glycerol/lactate in the presence of doxycycline failed to grow (Figure 6D). Thus, our observations suggest that depletion of *MAS1* or *MAS2* perturbs mitochondrial function in *C. albicans*.

### Ydj1 is important for mitochondrial import and function

**Figure 7:**
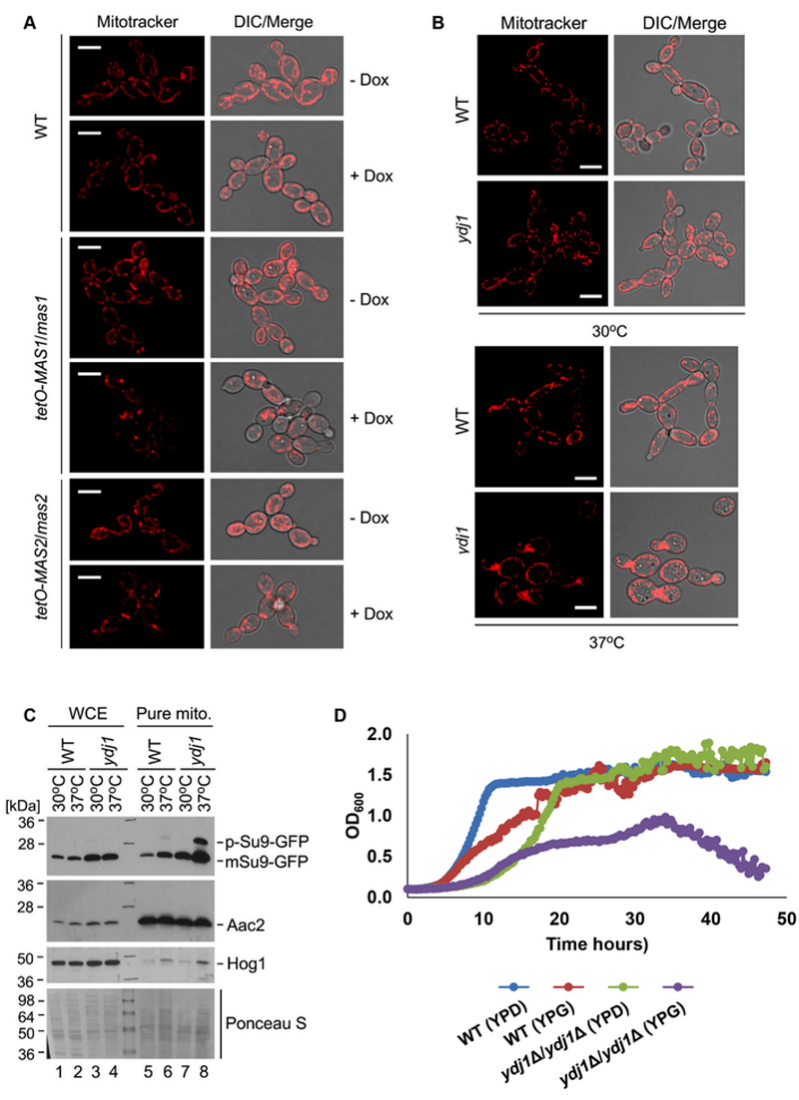
FIGURE 7: Mas1, Mas2 and Ydj1 are important for mitochondrial morphology and function. **(A)** The wild-type, *tetO-MAS1/mas1*Δ and *tetO-MAS2/mas2*Δ strains were grown in YPD in the absence (- Dox) or presence (+ Dox) of 20 μg/mL doxycycline for 24 hours, and then subcultured in the same conditions for an additional 4 hours to deplete *MAS1* and *MAS2*. Cells were stained with MitoTracker Red dye (mitochondria) for one hour, and examined by confocal microscopy. Representative images are shown. Scale bar indicates 5 μm. **(B)** Ydj1 is required for normal mitochondrial morphology at high temperatures. The *ydj1*Δ/*ydj1*Δ mutant was grown in YPD for 16 hours at 30°C, and then diluted 10 times in fresh YPD medium and grown for a further 6 hours at 30°C or 37°C. Samples were stained with 100 nM MitoTracker Red for 1 hour and examined by confocal microscopy. Representative images are shown. The scale bars indicate 5 μm. **(C)** Ydj1 is required for import of the artificial substrate Su9-GFP under elevated temperatures. Whole cell extract (WCE) and gradient-purified mitochondria were obtained from wild-type and *ydj1*Δ/*ydj1*Δ cells harboring *tetO-Su9-GFP*, which were grown at 30°C or 37°C for 6 hours. The blots demonstrate significant accumulation of the Su9-GFP precursor form in a *ydj1*Δ/*ydj1*Δ mutant grown at elevated temperatures. The anti-Hog1 and anti-Aac2 antibody staining was used as a marker of mitochondrial preparation purity. Ponceau S staining was used as control for protein loading. **(D)** Growth kinetics of the wild-type (SN95) laboratory strain of *C. albicans* and the *ydj1*Δ*/ydj1*Δ mutant were measured in YPD or YPG medium at 30°C with orbital shaking, with measurements taken every 15 minutes for 48 hours. P ≤ 0.05, Two-Way ANOVA, Sidak’s Multiple Comparisons Test.

To determine if Ydj1 plays any role in mitochondrial function in *C. albicans*, we first examined mitochondrial morphology. First, we grew wild-type, *tetO-MAS1/mas1*Δ and *tetO-MAS2/mas2*Δ strains in YPD in the presence or absence of doxycycline for 24 hours and then subcultured for an additional 4 hours under the same conditions with the addition of mitochondria-specific vital fluorescent dye Mito Tracker Red. Generally, mitochondria form dynamic tubular networks capable of changing shape and moving throughout the cell. Doxycycline-mediated transcriptional repression of *MAS1* or *MAS2* induced punctate, aggregated mitochondrial structures, reflecting dysfunction-induced fragmentation of the mitochondrial network (Figure 7A). Second, we looked at the *ydj1*Δ/*ydj1*Δ mutant strains in YPD with MitoTracker Red. At 30°C, the *ydj1*Δ/*ydj1*Δ mutant presented similarly to the wild type, with only a few punctate, aggregated mitochondrial structures (Figure 7B). Given that the severity of mitochondrial import phenotypes can be exacerbated at elevated temperature 14, we monitored mitochondrial network morphology at 37°C. At 37°C, we observed significant differences in the network morphology in the *ydj1*Δ/*ydj1*Δ mutant compared to wild-type cells (Figure 7B). The *ydj1*Δ/*ydj1*Δ mutant cells no longer contained tubular mitochondrial networks, but instead mitochondria were punctate, and often aggregated at the septum of the cell (Figure 7B). Thus, Ydj1 is required for normal mitochondrial morphology at elevated temperatures.

Next, to more explicitly test whether Ydj1 influences the import of mitochondrial proteins, we used the reporter construct comprising targeting signal (amino acid residues 1-69) of the ATP synthase subunit 9 (Su9) from *Neurospora crassa* and the GFP moiety 49. Su9(1-69) is a well characterised mitochondrial matrix targeting sequence which includes a processing site for the MPP Mas1 and Mas2, and three amino acids of the mature form of Su9 50. We hypothesised that in a strain lacking Ydj1, the precursor form of Su9-GFP (predicted to be 37 kDa), would accumulate, as Ydj1 is required to deliver proteins to Mas1 and Mas2 for cleavage. However, in wild-type cells, the precursor form would be cleaved upon import into the mitochondria, leading to the mature form (predicted to be 29 kDa). Wild-type and *ydj1*Δ/*ydj1*Δ mutant strains with the Su9-GFP construct were grown at 30°C or 37°C, and proteins were extracted from whole cells and pure mitochondria. Upon probing the whole cell extract for Su9-GFP, only the mature form was detected in both the wild type and *ydj1*Δ/*ydj1*Δ mutant (Figure 7C). However, the pure mitochondrial extracts revealed an accumulation of the precursor form of Su9-GFP in the *ydj1*Δ/*ydj1*Δ mutant at 37°C compared to wild type (Figure 7C). This is likely due to the fact that mitochondrial import phenotypes can be exacerbated at elevated temperature due to lower efficiency of substrate translocation 14. Thus, Ydj1 is required for full maturation of Su9-GFP, likely through its association with the MPP Mas1 and Mas2.

The data thus far suggests that Ydj1 plays a role in mitochondria import. As such, we hypothesised that mitochondrial function would be impaired in the *ydj1*Δ/*ydj1*Δ mutant. To test this, we assessed the growth of the wild-type and *ydj1*Δ/*ydj1*Δ mutant strains in YP-glucose (2%) and YP-galactose (2%), finding that the *ydj1*Δ/*ydj1*Δ mutant was unable to maintain sustained growth in YP-galactose (Figure 7D). Thus, Ydj1 is required for normal mitochondrial morphology, respiration and import of mitochondrial proteins.

### Ydj1 is localised to the cytosol and outer mitochondrial membrane

**Figure 8:**
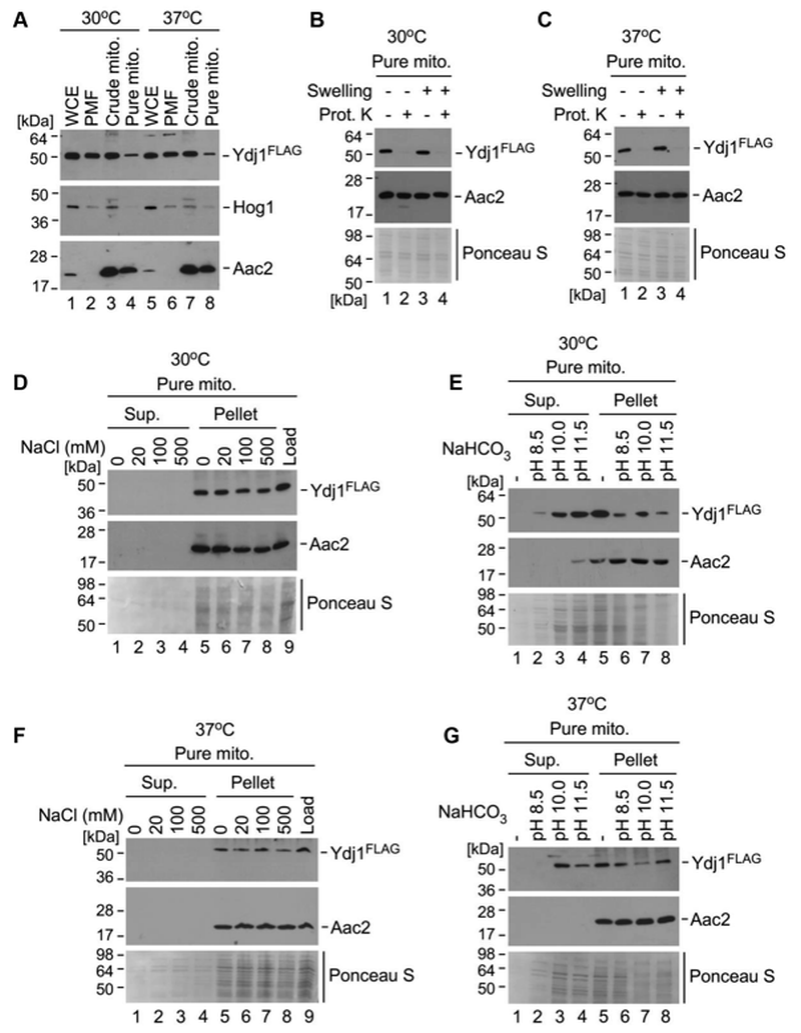
FIGURE 8: Mitochondrial fraction of Ydj1 is tightly associated with the outer membrane. **(A**) A fraction of Ydj1 is localised to mitochondria. WCE, PMFs and mitochondria isolated from 2×FLAG-*YDJ1/ydj1*Δ yeast cultures grown at 30^o^C and 37^o^C were analyzed by SDS-PAGE and immunoblotting with antibodies against the FLAG epitope, cytosolic protein Hog1 and the ADP/ATP mitochondrial carrier Aac2. Anti-FLAG antibody staining is present in all fractions suggesting pancellular localisation of Ydj1, which is expected. Ydj1 is also present in the gradient-purified mitochondrial fraction pointing at associations with mitochondria under normal (30^o^C) and elevated (37^o^C) temperatures. **(B **and** C)** Mitochondrial fraction of Ydj1 is associated with the outer membrane. Gradient-purified mitochondria isolated from 2×FLAG-*YDJ1/ydj1*Δ yeast cultures were subfractionated by osmotic disruption of the outer membrane (Swelling) combined with Proteinase K (Prot. K) treatment. Western blotting using anti-FLAG demonstrated Ydj1 localised to the outer mitochondrial membrane upon normal (30^o^C) and higher (37^o^C) temperature growth conditions. **(D **and** F)** Salt and **(E **and** G)** alkaline extractions of Ydj1 suggest tight peripheral association with the outer mitochondrial membrane. Western blotting analysis of salt extracts demonstrates presence of Ydj1 only in pelleted fractions isolated from gradient-purified mitochondria from the cells grown at both 30°C and 37°C. Positive antibody staining for Ydj1 appeared in Western blots which analysed the supernatants obtained from protein extracts under indicated alkaline conditions. Anti-serum against transmembrane protein Aac2 was used as a marker of integral membrane association. Ponceau S staining of the blots was used to visualise protein loads.

In *S. cerevisiae*, the majority of Ydj1 is found in the cytoplasm and endoplasmic reticulum, with a small fraction localising to mitochondria 13. Given the interaction of Ydj1 with the MPPs, which are mitochondrial proteins, and the phenotypes we have observed with defective mitochondrial morphology, import and growth on non-fermentable carbon sources, we proposed that Ydj1 does, in part, localise to the mitochondria in *C. albicans*. To assess this, we probed for Ydj1 from whole cell extracts, post-mitochondrial fractions, crude mitochondria, and pure mitochondria isolated from the FLAG tagged Ydj1 strain grown at 30°C and 37°C. Ydj1 was present in all fractions (Figure 8A), suggesting that it is present in the cytosol and mitochondria. However, the levels of Ydj1 in the pure mitochondrial fraction were lower than the levels of Ydj1 seen in all other subcellular fractions.

To determine the mitochondrial compartment to which Ydj1 localises, gradient purified mitochondria were mock treated or subjected to osmotic disruption (Swelling) of the outer membrane, followed by addition of the exogenous Proteinase K. Addition of Proteinase K causes a loss of Ydj1 in swollen and mock-treated mitochondria isolated at both 30°C (Figure 8B) and 37°C (Figure 8C), suggesting an association with the cytosolic side of the outer membrane. To assess this association further, we performed subcellular fractionations of Ydj1-containing mitochondria under salt and alkaline conditions. Western blotting analysis of salt extracts demonstrates the presence of Ydj1 and the control inner membrane-anchored protein Aac2 only in pelleted fractions isolated from gradient-purified mitochondria from the cells grown at both 30°C and 37°C (Figure 8D and 8F), indicating that Ydj1 is firmly bound to the outer mitochondrial membrane. Conversely, immunoblotting analysis of alkaline extracts revealed the majority of Ydj1 signal - but not Aac2 signal - in the supernatant fractions (Figure 8E and 8G), reflecting the peripheral association of Ydj1 with mitochondria. Taken together, these data suggest that a pool of Ydj1 is specifically and tightly associated with the cytosolic side of the outer mitochondrial membrane.

## DISCUSSION

As mitochondria evolved, the mitochondrial genome was reduced and has almost completely been incorporated into the nuclear genome. As such, most mitochondrial proteins are synthesised in the cytosol with a cleavable N-terminal sequence that targets them for import with the assistance of molecular chaperones. To date, the identity and function of mitochondrial import proteins in *C. albicans* has remained largely unknown. We identified a novel role for the *C. albicans* Hsp40 chaperone Ydj1 in mitochondrial import, implicating the mitochondrial peptidases Mas1 and Mas2 as central to this process.

Chaperones are fundamental to orchestrating protein trafficking and maintaining protein homeostasis during stress in the eukaryotic kingdom 51. For example, Hsp90 promotes the folding and function of key substrate proteins 24,52,53, often in collaboration with co-chaperones 54. Hsp90 has been well studied in *C. albicans* 22,24,26,55, but the roles of other chaperones remain largely unknown. For example, the Hsp40 chaperone Ydj1, the most studied Hsp40 chaperone in *S. cerevisiae*, had not been characterised in *C. albicans*. Utilising genetic and biochemical approaches, we have uncovered a plethora of diverse phenotypes related to stress responses upon loss of Ydj1 (Figure 1). These phenotypes may be attributable to the role of Ydj1 in promoting protein homeostasis. Consistent with this, our proteomic analysis revealed that Ydj1 interacts with eight other chaperones or co-chaperones, some of which have been previously characterised in *S. cerevisiae*, corroborating our findings. Indeed, a strong interaction was observed between Ydj1, Hsp104, Ssa2 and Hsp70, similar to findings in *S. cerevisiae*, where Ydj1 interacts with Hsp104 and Ssa1, together aiding in protein re-folding 41. Hsp104 and Hsp70 are both required for high temperature growth and virulence in *C. albicans* 56,57,58. Thus, the increase in stress sensitivity in the *C. albicans* *ydj1*Δ/*ydj1*Δ mutant could be due to an accumulation of misfolded proteins and a deficiency in re-folding aggregated proteins that occur during stress.

**Figure 9:**
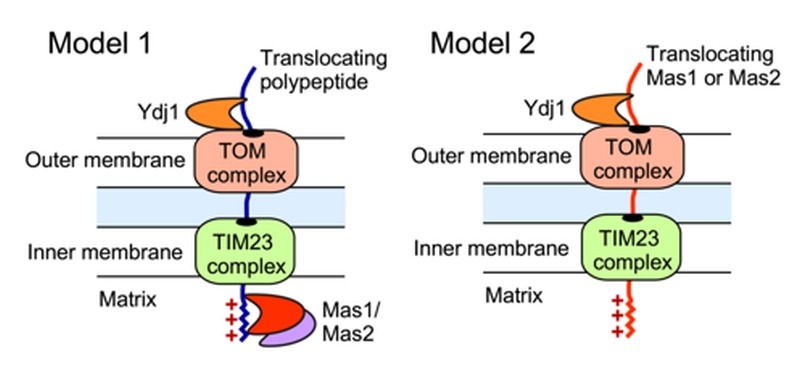
FIGURE 9: Schematic illustrating two possible models of Ydj1 interaction with the MPPs. Such interactions might be indirect via binding of Ydj1 to the protein substrates processed by MPP (model 1), or direct upon the translocation of either Mas1 or Mas2 to the mitochondria (model 2).

The impact of Ydj1 on cellular stress responses may also be mediated through control of mitochondrial function, which is crucial for cellular stress survival 27, and is contingent upon the proper import of proteins with a targeting signal sequence. We detected a strong interaction between Ydj1 and the MPP Mas1 and Mas2, both in the presence and absence of heat shock (Figure 3). In *S. cerevisiae*, Ydj1 functions together with Ssa1 to mediate the import of mitochondrial preproteins 14, but the precise mechanisms of targeting and import of mitochondrial proteins remains elusive. Mitochondria contain transport machineries in both the outer (TOM) and inner (TIM) membranes to import nuclear-encoded proteins, whereby they are cleaved by MPP 59. We established that Ydj1 is tightly associated with the mitochondrial outer membrane (Figure 8), and that Ydj1 from gradient purified mitochondria physically interacts with the MPP Mas1 and Mas2 (Figure 5A). Furthermore, *ydj1*Δ/*ydj1*Δ mutant cells displayed defective mitochondrial morphology and were unable to sustain growth on media containing a non-fermentable carbon source, suggesting that Ydj1 plays a role in mitochondrial function (Figure 7B and 7D). Utilising the model mitochondrial import protein, Su9-GFP, which is processed by Mas1 and Mas2 in the mitochondria, we found that deletion of *YDJ1* leads to an accumulation of the immature form of Su9-GFP at 37°C (Figure 7C). Thus, Ydj1 may interact with the MPPs indirectly, via binding with the MPPs substrate proteins during their import, or it may be a direct interaction, binding the MPP subunits upon their own translocation to the mitochondria (Figure 9). Further analysis with other MPP substrate proteins would be required to resolve the nature of the interaction.

We found that Ydj1 also interacts with other key players involved in mitochondrial import and function, including Hsp70, Mdj1 and Hsp78. Cytosolic Hsp70 proteins were the first chaperones implicated in mitochondrial protein import 60, with studies in yeast utilising synthetic peptides revealing that presequence peptides can bind cytosolic Hsp70 61. The mitochondrial DnaJ (Mdj1) is a soluble mitochondrial matrix protein that binds precursor proteins entering the matrix in the latter stages of import, aiding in their folding 62. Similar to Mdj1 is the soluble mitochondrial matrix protein Hsp78, which binds misfolded polypeptides in the matrix to prevent aggregation 63. We observed an interaction between Ydj1 and Hsp78 during heat shock, which impairs mitochondrial protein synthesis 63, suggesting that Ydj1, together with Hsp78 promotes re-folding of damaged proteins upon heat shock. Each of these chaperones are regulated by the heat shock transcription factor Hsf1 in *C. albicans *26. In *S. cerevisiae,* Hsf1 is required for import of mitochondrial proteins, likely attributable to regulatory control of Ydj1 and other chaperones 7,8. Thus, Ydj1 may promote survival in response to stress through interactions with co-chaperones, thereby promoting protein re-folding and mitochondrial function, which are both required for survival in response to cellular stresses 27.

As a consequence of their biochemical function in modulating protein homeostasis, molecular chaperones also have central roles in orchestrating temperature-dependent developmental programs. Temperature regulates numerous cellular processes in *C. albicans*, including a morphological transition from the yeast form at ambient temperatures to filamentous forms in response to thermal stress; this transition is important for dissemination, tissue penetration, immune evasion, and virulence 18. The expression of many molecular chaperones increases in response to elevated temperature 64, and the molecular chaperone Hsp21 serves as a regulator of morphogenesis 38. Thus, we hypothesised that Ydj1 might interact with and promote folding of a positive regulator of filamentous growth. Although none of the interactors we identified were strictly required for morphogenesis, as was Ydj1, we detected an interaction with three proteins that promote robust filamentation; the small heat shock protein Hsp21 38, as well as Cdc48 and Mas1, proteins both involved in mitochondrial function 5,45 (Figure 4). Notably, the interaction of Ydj1 with Hsp21 and Cdc48 increased with heat shock, suggesting that the regulation of temperature-dependent filamentation may occur in part through these regulators. Given that inhibition of mitochondrial function blocks *C. albicans* morphogenesis 32, and Mas1 and Cdc48 are both important for mitochondrial function, an alternative model is that the morphogenetic defect observed in the absence of Ydj1 may be attributable to reduced mitochondrial protein import. Thus, as a central regulator of mitochondrial function, cellular stress responses, and virulence traits in a leading fungal pathogen, Ydj1 provides a novel prospective target for antifungal drug development.

## MATERIALS AND METHODS

### Strains and growth conditions

All strains are listed in Table 1. Strains were grown in YPD (1% yeast extract, 2% bactopeptone, 2% glucose) or YPGD (1% yeast extract, 2% bactopeptone, 0.2% glucose, 2% galactose) 65 with shaking at 200 rpm. Doxycycline (BD Biosciences) was added to YPD medium at a concentration of 0.05 µg/ml or 20 µg/ml, as stated. MitoTracker Red (Life Technologies, M-7512) was added at 100 nM. To repress expression of *MAS1* or *MAS2* from the tetracycline-repressible (*tetO*) promoter, overnight cultures in YPD at 30°C were diluted to OD_600_ 0.2 in the absence or presence of doxycycline and cultured for 24 hours. Cells were diluted to OD_600_ 0.05 in the same conditions and grown to mid-log phase (~5 hours) prior to microscopy, RNA extraction, or Western blot analysis. A 30°C - 42°C heat shock was imposed, as indicated, following our established protocols 21.

**Table 1:** *C. albicans* strains.

Strain	Genotype	**Source**
SN95	*arg4∆/arg4∆ his1∆/his1∆ URA3/ura3∆::* λ*imm434* *IRO1/iro1::*λ*imm434*	66
CaLC3160 *ydj1*Δ/Δ	*arg4∆/arg4∆ his1∆/his1∆ URA3/ura3∆::* λ*imm434* *IRO1/iro1::*λ*imm434 ydj1/ydj1::FRT*	This study
CaLC3924 *ydj1*Δ/*YDJ1-ARG4*	*arg4∆/arg4∆ his1∆/his1∆ URA3/ura3∆::* λ*imm434* *IRO1/iro1::*λ*imm434 ydj1/YDJ1-ARG4*	This study
CaLC3958 FLAG-*YDJ1*/*ydj1*Δ	*arg4∆/arg4∆ his1∆/his1∆ URA3/ura3∆::* λ*imm434* *IRO1/iro1::*λ*imm434 ydj1/2xFLAG-YDJ1-ARG4*	This study
CaLC4390 *MAS1/mas1*Δ	*arg4∆/arg4∆ his1∆/his1∆ URA3/ura3∆::* λ*imm434* *IRO1/iro1::*λ*imm434 MAS1/mas1::FRT*	This study
CaLC4391 *MAS2/mas2*Δ	*arg4∆/arg4∆ his1∆/his1∆ URA3/ura3∆::* λ*imm434* *IRO1/iro1::*λ*imm434 MAS2/mas2::FRT*	This study
CaLC4410 *tetO-MAS1/mas1*Δ	*arg4∆/arg4∆ his1∆/his1∆ URA3/ura3∆::* λ*imm434* *IRO1/iro1::*λ*imm434 mas1::FRT CaTAR-FRT::tetO-MAS1*	This study
CaLC4438 *tetO-MAS2/mas2*Δ	*arg4∆/arg4∆ his1∆/his1∆ URA3/ura3∆::* λ*imm434* *IRO1/iro1::*λ*imm434 mas2::FRT CaTAR-FRT::tetO-MAS2*	This study
CaLC4392 *FLAG-YDJ1/ydj1*Δ-*MAS1/mas1*Δ	*arg4∆/arg4∆ his1∆/his1∆ URA3/ura3∆::* λ*imm434* *IRO1/iro1::*λ*imm434 ydj1/2xFLAG-YDJ1-ARG4/ MAS1/mas1::FRT*	This study
*tetO-CDC48/cdc48*Δ	*tetO-CDC48/cdc48*Δ	44
*tetO-TBP1/tbp1*Δ	*tetO-TBP1/tbp1*Δ	44
CaLC4422 *ACT1p-Su9-GFP-NAT/ACT1*	*arg4∆/arg4∆his1∆/his1 ∆URA3/ura3∆::imm434 IRO1/iro1::imm434* *ACT1/ACT1pSnSu9-GFP-NAT*	This study
CaLC4547 *tetO-CAS5/CAS5*	*arg4∆/arg4∆ his1∆/his1∆ URA3/ura3∆::imm434 IRO1/iro1::imm434* *CaTAr-FRT-tetO-CAS5/CAS5*	This study
CaLC4554 *tetO-CAS5/CAS5 ydj1*Δ/*ydj1*Δ	*arg4∆/arg4∆ his1∆/his1∆ URA3/ura3∆::imm434 IRO1/iro1::imm434* * ydj1∷FRT*/*ydj1∷FRT CaTAr-FRT-tetO-CAS5/CAS5*	This study
CaLC4562 *tetOp-Su9-GFP-NAT/CAS5*	*arg4∆/arg4∆ his1∆/his1∆ URA3/ura3∆::imm434 IRO1/iro1::imm434* *CaTAr-FRT-tetO-SnSu9-GFP-NAT/CAS5*	This study
CaLC4611 *tetOp-Su9-GFP-NAT/CAS5 ydj1*Δ/*ydj1*Δ	*arg4∆/arg4∆his1∆/his1∆URA3/ura3∆::imm434 IRO1/iro1::imm434* *ydj1::FRT*/*ydj1::FRT CaTAr-FRT-tetO-SnSu9-GFP-NAT/CAS5*	This study
CaLC4967 *FLAG-YDJ1/MAS1-MYC*	*arg4∆/arg4∆ his1∆/his1∆ URA3/ura3*∆*::imm434 IRO1/iro1::imm434* *FLAG-YDJ1-ARG/ydj1∷FRT MAS1-MYC-NAT/MAS1*	This study
CaLC4969 *FLAG-YDJ1/MAS2-MYC*	*arg4∆/arg4∆ his1∆/his1∆ URA3/ura3∆::imm434 IRO1/iro1::imm434* *FLAG-YDJ1-ARG/ydj1∷FRT MAS2-MYC-NAT/MAS2*	This study

### Strain construction

To generate an *YDJ1* homozygous deletion mutant, the NAT flipper cassette (pLC49, Table 2) 67 was PCR amplified with oLC3129/oLC3131 containing sequence homologous to the upstream and downstream regions of *YDJ1*. The PCR product was transformed into the wild-type strain SN95 (CaLC239), and NAT-resistant transformants were PCR tested with oLC275/oLC3132 and oLC274/oLC3133 to verify integration of the cassette. The NAT cassette was excised, generating CaLC3124 (*YDJ1/ydj1*Δ). A second round of PCR was performed to delete the second allele by re-amplifying the NAT flipper cassette with oLC3128/oLC3130. The PCR product was transformed into CaLC3124 and NAT-resistant transformants were PCR tested with oLC275/oLC3132 and oLC274/oLC3133 to verify integration of the cassette, and oLC3134/oLC3135 to verify loss of the wild-type allele, creating CaLC3160 (*ydj1*Δ/*ydj1*Δ).

**Table 2:** Plasmids used in this study.

Strain	Genotype	**Source**
pLC49	*FLP-CaNAT, ampR*	68
pLC605	*CaTAr-FLP-CaNAT, ampR*	69
pLC389	*GFP-NAT, ampR*	70
pLC578	*pFA-MYC-HIS1, ampR*	71

To regulate *MAS1 *and *MAS2*, one allele of *MAS1* or *MAS2* was deleted, and the other was placed under the control of the *tetO* promoter that can be repressed with tetracycline or the analog doxycycline. Briefly, the NAT flipper cassette (pLC49), containing sequence homologous to the upstream and downstream regions of *MAS1* or *MAS2* was PCR amplified with oLC4284/oLC4285 (*MAS1*) or oLC4295/4296 (*MAS2*). The PCR product was transformed into the wild-type strain SN95 (CaLC239), and NAT-resistant transformants were PCR tested with oLC275/oLC4286 and oLC274/oLC4283 (*MAS1*) or oLC275/oLC4297 and oLC274/oLC4294 (*MAS2*) to verify integration of the cassette. The NAT cassette was then excised to create CaLC4390 (*MAS1/mas1*Δ) or CaLC4391 (*MAS2/mas2*Δ). The tetracycline-repressible transactivator, the *tetO *promoter, and the NAT flipper cassette were PCR amplified from pLC605 (Table 2) 72 using oligos oLC4284/oLC4287 (*MAS1*) or oLC4295/oLC4298 (*MAS2*). The PCR product was transformed into CaLC4390 (*MAS1/mas1Δ*) or CaLC4391 (*MAS2/mas2*Δ). Correct integration at the *MAS1* locus was verified by colony PCR using primer pairs oLC534/oLC4286 and oLC4288/oLC300, respectively, generating CaLC4410 (*tetO-MAS1/mas1*Δ). Loss of the wild-type allele was verified with oLC4286/oLC4288. Correct integration for *MAS2* was verified using oligos oLC534/oLC4297 and oLC4299/oLC300. Loss of the wild-type allele was confirmed using primers oLC4297/4299, generating CaLC4438 (*tetO-MAS2/mas2*Δ).

To detect Ydj1 interactors by mass spectrometry, Ydj1 was tagged with 2xFLAG at its N-terminus. Our previous attempts to C-terminally tag Ydj1 resulted in a non-functional protein. The *YDJ1-ARG4* cassette was PCR amplified from CaLC3924 with long primers containing 2xFLAG (oLC3940/3807). The subsequent product was PCR amplified using primers oLC3941/3807, which contain homology upstream and downstream of the *YDJ1* locus, and transformed into the *ydj1*Δ/*ydj1*Δ strain (CaLC3160). Arginine prototrophic transformants were PCR tested for upstream integration using oLC3132/3828 and downstream integration using oLC3938/1594, generating CaLC3958 (*FLAG-YDJ1/ydj1*Δ). This strain also acted as a complemented strain for the *ydj1*Δ*/ydj1*Δ mutant.

To detect Mas1 and Mas2, the proteins were Myc tagged at their C-termini using a Myc-NAT cassette. The Myc-NAT cassette was generated using fusion PCR. Myc-tag with homology to *MAS1* or *MAS2* and NAT was PCR amplified from pLC578 (Table 2) with oLC5140 and oLC5157 (905 bp) for *MAS1* and oLC5142 and oLC5157 (905 bp) for *MAS2*. The NAT marker with homology to Myc and *MAS1* was PCR amplified from pLC49 with oLC5156 and oLC5158 (977 bp). The NAT marker with homology to Myc and *MAS2* was PCR amplified from pLC49 with oLC5156 and oLC5159 (977 bp). The PCR product was purified by spin column purification and 1:100 dilution was used as templates for the fusion PCR at a 1:1 molar ratio (~0.4 ng of the longer template). For Mas1, the Myc-NAT cassette was amplified with oLC5140 and oLC5158 and transformed into CaLC3958. Correct integration at the C-terminus of *MAS1* was verified by amplifying across both junctions using primer pairs oLC4282 + oLC2029 (upstream 632 bp) and oLC4283 + oLC3849 (downstream 955 bp), generating CaLC4967. For Mas2, the Myc-NAT cassette was amplified with oLC5142 and oLC5159 and transformed into CaLC3958. Correct integration at the C-terminus of *MAS2* was verified by amplifying across both junctions using primer pairs oLC4293 + oLC2029 (upstream 783 bp) and oLC4294 + oLC3849 (downstream 1106 bp), generating CaLC4969.

The *ACT1p-SnSu9-GFP-NAT* cassette was generated using fusion PCR. *ACT1p-SnSu9* was PCR amplified from the Su9-GFP plasmid, pYX142 49, using primers oLC4348/oLC4349. The GFP-NAT cassette was PCR amplified from pLC389 (Table 2) using primers oLC4350/oLC4275. The PCR products were spin column purified and the concentration was measured by nanodrop. The products were diluted 1:20 and added to the PCR mastermix in 1:1 molar ratio (~15 ng of the longer PCR product). The fusion was PCR amplified with oLC4348/oLC4275 and transformed into CaLC239 (SN95), generating CaLC4422 (*ACT1p-Su9-GFP-NAT/ACT1*). Correct integration at the *ACT1* locus was verified by PCR using primer pairs oLC3833/oLC600 and oLC274/oLC3024.

The *tetO-SnSu9-GFP-NAT* cassette was made by PCR amplifying the genomic DNA of CaLC4422 using oLC4384/oLC2644. The PCR product was transformed into CaLC4547 (*tetO-CAS5/CAS5*) to target integration at the *CAS5* locus, generating CaLC4562 (*tetOp-Su9-GFP-NAT/CAS5*). Correct integration at the *tetO-CAS5 *locus was verified by PCR amplifying across both junctions using primer pairs oLC2034/oLC534, oLC300/oLC600 and oLC274/oLC2164. CaLC4547 was generated by PCR amplifying the tetracycline-repressible transactivator, *tetO *promoter and NAT flipper cassette from pLC605 using primers oLC2088/oLC2089 and transformed into CaLC239. Correct upstream and downstream integration at the *CAS5 *locus was verified by PCR amplifying across both junctions using primer pairs oLC2034/oLC534 and oLC300/oLC2145.

To express Su9 in the *ydj1*Δ/*ydj1*Δ mutant, first the tetracycline-repressible transactivator and *tetO *promoter were PCR amplified from pLC605 using primers oLC2088/oLC2089 and transformed into CaLC3160 (*ydj1*Δ/*ydj1*Δ), generating CaLC4554 (*ydj1*Δ*/ydj1*Δ *tetO-CAS5/CAS5*). Correct upstream and downstream integration at the *CAS5 *locus was verified by PCR using primer pairs oLC2034/oLC534 and oLC300/oLC2145. Second, the *tetO-SnSu9-GFP-NAT* cassette was PCR amplified from the genomic DNA of CaLC4422 using oLC4384/oLC2644 and transformed into CaLC4554. Correct integration at the *tetO-CAS5 *locus was verified by PCR amplifying across both junctions using primer pairs oLC2034/oLC534 and oLC274/oLC2164. All primers are listed in Table 3.

**Table 3:** Primers.

Number	Gene	Sequence
oLC3128	CaYDJ1-NAT1 F1	CTTTTTTCCCTTCTTGTTTTTCATATCCACCCACATATTTATTTGCACATTAGATTTATAGAGTAGGAAACAGCTATGACCATG
oLC3129	CaYDJ1-NAT1 F2	GATAATTCAAAACGTCCAAAAGAACTTAAATTGGTTAATCATTATTAATATTTTTATCAGATCCCAACCGGAAACAGCTATGACCATG
oLC3130	CaYDJ1-NAT1 R1	CTTAATTACAATACTATTATTCACAAAATATCACTTTATATATATATATGATAATGATACTGCATAATGTAAAACGACGGCCAG
oLC3131	CaYDJ1-NAT1 R2	GAGTCTTATTGCTCGATGTGCTTGCCAGTAAAGTTAACCACTACCCCTATGGACAGTACCTACAAGGTAAAACGACGGCCAG
oLC3132	CaYDJ1d Ext F (-315 bp)	GAAGCTATCTTGTAAGACAG
oLC3133	CaYDJ1d Ext R (+1483 bp)	GCCTTTAACTAGAAGATAC
oLC3134	CaYDJ1d Int F (+170 bp)	GAGTGATGACCAAAAAAGAG
oLC3135	CaYDJ1d Int R (+809 bp)	GCCAATTCAATCTCTTGTTC
oLC274	pJK863down-F	CTGTCAAGGAGGGTATTCTGG
oLC275	pJK863up-R	AAAGTCAAAGTTCCAAGGGG
oLC4282	CaMAS1 +1122 Int F	GATGAAGAGGTAGAAAGATC
oLC4283	CaMAS1 +1728 Ext R	CAACAAGCACTTAATAGTGG
oLC4284	CaMAS1-pLC49 F	AGTAATCATTTATCACTCACATTGAATACTGTCTTCTTTCTTTTTGATTAACCAACAAAAACTCAAAAATGGAAACAGCTATGACCATG
oLC4285	CaMAS1-pLC49 R	TTATGTGTATATTCAAGGTATGCTTATTAGTCTTTAGTGAGTAGTTTTTGATATGTACAAGATAAACAAAGTAAAACGACGGCCAG
oLC4286	CaMAS1 -235 Ext F	CTGGTCATTTTTTGTCTCTC
oLC4287	CaMAS1 TetO pLC605 R	CAGCAGTGTTGAAACCACGATACTTTATACCACCGTTGTATTTCTTCAATGCACTAAGTCGTTTAAACATcgactatttatatttgtatg
oLC4288	CaMAS1 +493 Int R	CTTCATCGTACATTTTGTCG
oLC4293	CaMAS2 +1136 Int F	GTTAGTCACAGAAGAATCAC
oLC4294	CaMAS2 +2044 Ext R	GTTGATACATTATCCAAGGC
oLC4295	CaMAS2-pLC49 F	TTTTGTTTTCAACTCCATTAATCAAGTTTAACCTTTTTTTTTTTTACAAGTTTCAAGACATCCACCATCAGGAAACAGCTATGACCATG
oLC4296	CaMAS2-pLC49 R	TCTATGTGTATCTATTATAATTTTTTGGTGTAAATACATGTATATATACAAGAATGGTATTCCACTTCATGTAAAACGACGGCCAG
oLC4297	CaMAS2 -230 Ext F	GCTGAGTGTGTGACATATAC
oLC4298	CaMAS2 TetO pLC605 R	TTGCAAATGATAGTAAAGATTTTGAAACACCTTTATTTAGTTTCCTAGTTATTGTTCTTCTACTTAGCATcgactatttatatttgtatg
oLC4299	CaMAS2 +473 Int R	GCTTCTTGGAATTCTTGATC
oLC534	CaTAR-797-R	GATGGAGATAGTTTACGG
oLC300	Tetp-F-NotI	ATAAGAATGCGGCCGCGTTTGGTTCAGCACCTTGTCG
oLC600	JB-GFP+344-R	CCTTCAAACTTGACTTCAGC
oLC3940	2XFLAG-CaYDJ1-F	CATTATGGATTATAAAGATGATGATGATAAAGGTGGTGATTATAAAGATGATGATGATAAAGGTGGTGTTAAAGACACAAAGTTTTAC
oLC3941	CaYDJ1-FLAG F	CTTTTCTTTTTTCCCTTCTTGTTTTTCATATCCACCCACATATTTATTTGCACATTAGATTTATAGAGTAATGGATTATAAAGATGATGA
oLC3807	CaYDJ1 TAP-ARG4 R	TGCTTAATTACAATACTATTATTCACAAAATATCACTTTATATATATATATGATAATGATACTGCATAATtcgatgaattcgagctcgtt
oLC3938	CaYDJ1+1631 R	CGCAATAGTGATTTCTGATC
oLC3828	CaYDJ1+333 R	GATGTCTTTACCTCTAGATG
oLC1594	ARG4-F	ATGTTGGCTACTGATTTAGCTG
oLC4348	ACT1p-NcSu9-F	ACTCCTGGTTTTCTTTCTTTCTTAGAAACATTATCTCGATATTAATATTAAAAAAATATAATCATTCAAAATGGCCTCCACTCGTGTCCT
oLC4349	NcSu9-GFP-R	ATAATTCTTCACCTTTAGACATGGTGGCGATGGATCCGG
oLC4350	NcSu9-linker-GFP-F	CCGGATCCATCGCCACCATGTCTAAAGGTGAAGAATTAT
oLC4275	CaACT1t pLC389_R	ATGTAATAACAAAAAGAAGAATAACAAGAATACAAAACCAGATTTCCAGATTTCCAGAATTTCACTCGTAAAACGACGGCCAGTGAATTC
oLC3833	CaACT1-624-F	GTGAAGCAAGTATATCTGGC
oLC3024	CaACT1+2493-R	GCTGTAAAACAAACAACTCG
oLC4384	tetO-Su9_pLC605 F	TCAACTACAATAAAACCAACTACGTCTACTACTACGTTAACTCCTACACACATACAAATATAAATAGTCGATGGCCTCCACTCGTGTCCT
oLC2644	GFP-CaCAS5-R	ATACGAATTATCTATATGGATTATACTTTAAATAATACCGTCTTTTAATGCATAGTCTATATAATGTGTAAAACGACGGCCAGTGAATTC
oLC2034	CaCas5-465F	GCTTGGATTTTCCCCCATTAG
oLC2088	CaCAS5_pLC605F	CTATTCTAATTTATTTACTTTGCTTTTCATCCCACCCCTTTGTTGGTAAATATAGACTTTAACATATACTGGAAACAGCTATGACCATG
oLC2089	CaCAS5_pLC605R	GTAAACTATTTGTACCATCATCATATGGCTGTGATAGCTGTGTCGGCGAACTTAATAAATAATTCTCCATcgactatttatatttgtatg
oLC2145	CaCAS5+169-R	GGCTTGCAGTTGTACTTGC
oLC534	CaTAR-797-R	GATGGAGATAGTTTACGG
oLC2164	CaCAS5+2737R	GTCATCATGCGAGTTATGG
oLC4376	CaMAS2 +240F	CCAGGATTATCTTATTTGCG
oLC4377	CaMAS1 +219F	CACTTTTTAGAGCATTTGGC
oLC2285	CaACT1+855-F	GACCTTGAGATACCCAATTG
oLC2286	CaACT1+1076-R	CAGCTTGAATGGAAACGTAG
oLC5140	CaMAS1+1332-F pLC578	AGCTTTGGCTGCAGTGGGGAACGTGAAGACCTTACCTTCACACAAAGAAATCAGCGAAGGAATGTTTTTCCCCGGGGAACAGAAGCTTAT
oLC5157	MYC-NAT-R	CAATAGCTTCAGCATCACCTGGAACAGAAGTTCTGTATCTATAAGCAGTATCATCCAAAGTAGTAGACATTATCGGTAGTTGGTGGTTAA
oLC5156	MYC-NAT-F	TTTTTATTTTTTTTTGGTGAAGATTTTTCCCACACAACTTCTTCTTTTACTTAACCACCAACTACCGATAATGTCTACTACTTTGGATGA
oLC5158	CaMAS1+1474-R NAT	TTATGTGTATATTCAAGGTATGCTTATTAGTCTTTAGTGAGTAGTTTTTGATATGTACAAGATAAACAAAACTGGATGGCGGCGTTAGTA
oLC2029	CaHA-R	GGCGAGGTATTGGATAGTTC
oLC3849	CaNAT+187-F	GATGAATCCGATGATGAATC
oLC5142	CaMAS2+1497-F pLC578	TCCAGAAATCACGGAACCAAGAGATTTCAGTAGTAATCAAAATGAAAAGAAGAAAAAGAGTCGTTGGTTTCCCGGGGAACAGAAGCTTAT
oLC5157	MYC-NAT-R	CAATAGCTTCAGCATCACCTGGAACAGAAGTTCTGTATCTATAAGCAGTATCATCCAAAGTAGTAGACATTATCGGTAGTTGGTGGTTAA
oLC5159	CaMAS2+1639-R NAT	TCTATGTGTATCTATTATAATTTTTTGGTGTAAATACATGTATATATACAAGAATGGTATTCCACTTCATACTGGATGGCGGCGTTAGTA

### Phenotypic assays

The susceptibilities of strains to stress were determined with a constant concentration of the following stressors: calcofluor white (CFW: 25 µg/ml), NaCl (1 M), H_2_O_2_ (5 mM), and temperature (30°C, 37°C, or 42°C). All stresses were assayed in flat-bottom 96-well microtiter plates (Starstedt) in a final volume of 200 µl/well. Overnight cultures were diluted in YPD to ~10^3^ cells/ml and used to inoculate the first well. Cells were subsequently diluted ten-fold in YPD in wells across the plate. Plates were wrapped in tin foil and incubated statically at 30°C (except for temperature screens) for 48 or 72 hours. Cells were re-suspended and final cell densities were determined by measuring OD_600_ using a spectrophotometer. Data was quantitatively plotted with colour using Java Treeview 1.1.3 (http://jtreeview.sourceforge.net). Assays were performed in duplicate on three different occasions.

### Minimum inhibitory concentration assay

Antifungal tolerance was determined in flat-bottom, 96-well microtiter plates (Starstedt). MICs were set up to a final volume of 200 µl/well with two-fold dilutions of caspofungin (Merck) and the last well containing no caspofungin. Overnight cultures were diluted in YPD such that ~103 cells were inoculated into each well. Plates were wrapped in tin foil and incubated at 30°C for 48 hours. Cells were re-suspended and final cell densities were determined by measuring OD_600_ using a spectrophotometer. Caspofungin tolerance was tested in duplicate on three different occasions. Data was quantitatively plotted with colour using Java Treeview 1.1.3.

### Microscopy

Filamentation was observed by subculturing overnight cultures of indicated strains into YPD medium in the absence or presence of 10% newborn bovine serum (Gibco #26010-066) at an OD_600_ 0.1 and growing cells at 30°C or 37°C for four hours. For high temperature filamentation, overnight cultures were subcultured into YPD at an OD_600_ 0.1 and grown with shaking at 30°C or 39°C for three hours. Imaging was performed on a Zeiss Imager M1 upright microscope and AxioCam MRm with AxioVision 4.7 software.

Mitochondrial morphology in the wild -type, *ydj1*Δ/*ydj1*Δ, *tetO-MAS1/mas1*Δ and *tetO-MAS2/mas2*Δ strains was assessed by subculturing an overnight culture of each strain into YPD at an OD_600_ 0.1 in the absence or presence of 20 μg/ml doxycycline for 24 hours. Strains were subcultured again in the same conditions with the addition of 100 nM MitoTracker and grown for a further four hours at 30°C or 37°C before imaging. Mitotracker Red-stained live cells were visualized as described before 29 using the Olympus IX81-FV5000 confocal laser scanning microscope at 543-nm laser line with a 100× oil lens. Images were acquired and processed with Fluoview 500 software (Olympus America).

### qRT-PCR

To ensure depletion of *MAS1* and *MAS2 *transcript levels, strains SN95 (CaLC2993), CaLC4410 (*tetO*-*MAS1/mas1*∆) and CaLC4438 (*tetO-MAS2/mas2*∆) were grown overnight at 30°C in YPD with shaking at 200 rpm. Stationary phase cultures were split, adjusted to an OD_600_ of 0.1 where one culture was treated with 20 μg/ml doxycycline, while the other remained untreated. Cells were grown for 24 hours at 30°C. After 24 hours, cultures were re-inoculated into YPD +/- doxycycline as per the previous growth condition. Cells were grown for 5 hours at 30°C and 10 ml was harvested from each culture, centrifuged at 3000 rpm for 2 minutes at 4°C, washed once with dH_2_O before being frozen at -80°C. RNA was subsequently isolated using the QIAGEN RNeasy kit and cDNA synthesis was performed using the AffinityScript cDNA synthesis kit (Stratagene). qRT-PCR was carried out using the Fast SYBR Green Master Mix (Thermo Fisher Scientific) in 384-wells with the following cycle conditions: 95°C for 3 minutes, 95°C for 10 seconds and 60°C for 30 seconds for 39 rounds, 95°C for 10 seconds, 65°C for 5 seconds. All reactions were performed in triplicate using the following primer pairs: *MAS1 *(oLC4377/4288), *MAS2 *(oLC4376/4299). Transcript levels were normalised to *ACT1* (oLC2285/2286). Data were analysed using the BioRad CFX Manager software, version 3.1 (BioRad).

### Growth assays

For the spot assays, wild-type (SN95), *tetO-MAS1/mas1*Δ and *tetO-MAS2/mas2*Δ strains were grown in the absence or presence of 20 μg/ml doxycycline in YPD for 24 hours. The cells were then serially diluted (with the lowest dilution being OD_600_ 1) and plated on YP-based plates containing glucose (2%), galactose (2%) or glycerol/lactate (each at 2%), with or without doxycycline. Plates were incubated at 30°C or 37°C for 36 hours and imaged. For the growth curves, overnight cultures of wild type or *ydj1*Δ*/ydj1*Δ grown in YPD at 30°C were subcultured into YPGD at an OD_600_ of 0.1 for 24 hours at 30°C. Growth kinetics were measured by inoculating cells from the cultures grown in YPGD at 30°C to an OD_600_ of 0.0625 in 100 µl of YP with 2% glucose or 2% galactose in flat bottom, 96-well microtiter plates (Sarstedt). Cells were grown in a Tecan GENios microplate reader (Tecan Systems Inc.) at 30°C with orbital shaking. Optical density measurements were taken every 15 minutes for 48 hours. Statistical significance was evaluated using GraphPad Prism 6.01.

### Western blotting

Proteins were extracted and subjected to Western blotting by pelleting cells at an OD_600_ 0.8 by centrifugation, washing with sterile H_2_O, and resuspending in 50 μl of 2x sample buffer (0.35 M Tris-HCl, 10% (w/w) SDS, 36% glycerol, 5% β-mercaptoethanol, and 0.012% bromophenol blue). Extracts were loaded in wells of a 6% or 10% SDS-PAGE gel. Separated proteins were transferred to a PVDF membrane for 1 hour at 100 V at 4°C. Membranes were blocked in 5% milk in PBS containing 0.1% Tween-20 at room temperature for 1 hour and subsequently incubated in primary antibody for one hour at room temperature in PBS-T+5% milk. Membranes were washed with 1x PBS-T and probed for one hour with secondary antibody dissolved in 1x PBS-T+5% milk (PBS, 0.1% Tween-20, 5% (w/v) milk). Membranes were washed in PBS-T and signals detected using an ECL Western blotting kit as per the manufacturer’s instructions (Pierce).

FLAG-tagged Ydj1 was detected using a 1:10,000 dilution of anti-FLAG HRP conjugated antibody (Sigma, A8592). To detect GFP a 1:1,000 dilution of anti-GFP (Santa-Cruz, sc-8334) was used. To detect Tubulin, an anti-Tub1 antibody was used (AbD Serotec, MCA78G) at a 1:5,000 dilution. To detect Myc, an anti-Myc (mouse) (11667149001, Roche Diagnostics) at 1:1,000 or anti-Myc (rabbit) (A-14, Santa Cruz Biotechnology) at 1:2,500 was used. To detect Aac2, an anti-Aac2 (rabbit) was used at a 1:5,000 dilution, a gift from Dr. Carla Koehler (UCLA). To detect Atp2, an anti-Atp2 (rabbit) serum was used at a 1:1,000 dilution, a gift from Dr. Alex Tzagoloff (Columbia University). Hog1 was detected using anti-Hog1 (y215, Santa Cruz Biotechnology) at 1:2,500. Secondary antibodies were anti-rabbit (7074S, Cell Signaling) at 1:5,000, and anti-mouse (115-035-068, Jackson ImmunoResearch Laboratories, Inc.) at 1:5,000.

### Ydj1 affinity purification

Untagged wild-type (SN95) and 2xFLAG-*YDJ1/ydj1*Δ (CaLC3958) cells were grown overnight at 30°C in YPD. Stationary phase cultures were split, adjusted to an OD_600_ of 0.1 in YPD and grown to an OD_600_ of 0.8. Cultures were split and subjected to a 15 minute 30°C - 42°C heat shock, exactly as previously described 21. Cells were harvested at 4000 rpm for 10 minutes at 4°C, washed twice with ice-cold 1x PBS and snap frozen in liquid nitrogen. A second biological replicate was performed on a separate date. One-step affinity purification of 2xFLAG-Ydj1 was performed using anti-FLAG M2 Magnetic Beads (Sigma-Aldrich), as previously described 73 with some modifications. Briefly, Pellets from 250 ml of cultured *C. albicans* yeast cells were resuspended in 10 ml of lysis buffer (100 mM HEPES, pH 8.0, 20 mM magnesium acetate, 10% glycerol (v/v), 10 mM EGTA, 0.1 mM EDTA, 0.4% NP-40 supplemented with fresh protease inhibitors mixture (100 fold dilution; P8215 (Sigma-Aldrich) and 1 mM PMSF), and subsequently lysed by bead-beating for 3 x 2 minutes in a cold room. The lysates were then sonicated for 3 x 30 second pulses at 65% amplitude using a QSONICA 125W sonicator equipped with 1/8” probe. The lysates were then centrifuged at 1,800 x*g* for 10 minutes and the resulting supernatant transferred to a fresh tube. Freshly washed anti-FLAG M2 magnetic beads (30 μl slurry per sample) were added and the samples were incubated with end-over-end rotation for 3 hours at 4°C. Using a DynaMag-2 magnet (ThermoFisher), the beads were collected on the side of the sample tube and the supernatant was discarded. The beads were washed three times by resuspension in 1 ml of wash buffer (100 mM HEPES, pH 7.4, 20 mM magnesium acetate, 10% glycerol (v/v), 10 mM EGTA, 0.1 mM EDTA, 0.5% Nonidet P-40) and transferred to a fresh 1.5 ml tube during the last wash. Finally, the beads were washed with 1 ml of 20 mM Tris-HCl pH8, 2 mM CaCl_2_. Following the last wash, the samples were quickly centrifuged and the last drops of liquid removed with a fine pipette. The now-dried beads removed from the magnet were resuspended in 7.5 µL of 20 mM Tris-HCl (pH 8.0) containing 750 ng of trypsin (Sigma-Aldrich; Trypsin Singles, T7575), and the mixture was incubated at 37°C with agitation overnight (~15 hours). After this first incubation, the sample was magnetized and the supernatant transferred to a fresh tube. Another 250 ng of trypsin was added (in 2.5 µL of 20 mM Tris-HCl (pH8)), and the resulting sample was incubated at 37°C for 3 - 4 hours without agitation. Formic acid was then added to the sample to a final concentration of 2% (from a 50% stock solution) and the samples were stored at -40°C until analyzed by mass spectrometry. Pellets prepared from untagged parental strains were used as negative controls and processed in parallel to Ydj1 for the AP-MS experiments.

### Mass spectrometry and data analysis

Ydj1 AP-MS samples and controls were analyzed by mass spectrometry in two biological replicates. 5 µl of each sample representing approximately 50% of the samples were directly loaded at 400 nl/min onto a 75 μm x 12 cm emitter packed with 3 µm ReproSil-Pur C18-AQ (Dr. Maisch HPLC GmbH.). Peptides were eluted from the column over a 90-minute gradient generated by a NanoLC-Ultra 1D plus (Eksigent) nano-pump and analyzed on a TripleTOF^TM^ 5600 instrument (AB SCIEX). The gradient was delivered at 200 nl/minute, starting at 2% acetonitrile (all solvents also contain 0.1% formic acid) and ending at 35% acetonitrile over 90 minutes, followed by a 15-minute clean-up in 80% acetonitrile, and a 15-minute equilibration period back to 2% acetonitrile for a total of 120 minutes. To minimize carryover between each sample, the analytical column was washed for 3 hours by running an alternating “saw-tooth” gradient from 35% acetonitrile to 80% acetonitrile, holding each gradient concentration for 5 minutes. Analytical column and instrument performance were verified after each sample by loading 30 fmol BSA tryptic peptide standard (Michrom Bioresources Inc.) with 60 fmol α-Casein tryptic digest and running a short 30 min gradient. TOF MS calibration was performed on BSA reference ions before running the next sample in order to adjust for mass drift and verify peak intensity. The instrument method was set to a Data Dependent Acquisition mode which consisted of one 250 ms MS1 TOF survey scan from 400 - 1300 Da followed by twenty 100 ms MS2 candidate ion scans from 100 - 2000 Da in high sensitivity mode. Only ions with a charge of 2+ to 4+ that exceeded a threshold of 200 cps were selected for MS2, and former precursors were excluded for 10 seconds after 1 occurrence.

Mass spectrometry data generated were stored, searched and analyzed using the ProHits laboratory information management system (LIMS) platform 74. Within ProHits, the initial WIFF files were first converted to an MGF format using WIFF2MGF converter and to an mzML format using ProteoWizard (v3.0.4468) and the AB SCIEX MS Data Converter (V1.3 beta) and then searched using Mascot (v2.3.02) and Comet (v2012.02 rev.0), respectively. The spectra were searched with the RefSeq database (version 68, November 21th, 2014) acquired from NCBI against a total of 29,524 *C. albicans* sequences supplemented with “common contaminants” from the Max Planck Institute (http://141.61.102.106:8080/share.cgi?ssid=0f2gfuB and the Global Proteome Machine (GPM; http://www.thegpm.org/crap/index.html . The database parameters were set to search for tryptic cleavages, allowing up to 2 missed cleavage sites per peptide with a mass tolerance of 40 ppm for precursors with charges of 2+ to 4+ and a tolerance of +/- 0.15 amu for fragment ions. Variable modifications were deamidated asparagine and glutamine and oxidized methionine. The results from each search engine were analyzed through the Trans-Proteomic Pipeline (TPP v4.6 OCCUPY rev 3) 75 via the iProphet pipeline 76. SAINTexpress (v3.3) 77 was used as a statistical tool to calculate the probability value of each potential protein-protein interaction from background contaminants using default parameters and a ProteinProphet cutoff of 0.95. Controls were kept uncompressed and a FDR of 1% or lower was required for proteins to be classified as significant interaction partners of Ydj1.

Dot plots and heat map of Ydj1 interaction networks obtained by AP-MS were generated using a web-based custom-built tool 78. All RAW mass spectrometry data and downloadable identification and SAINTexpress results tables are deposited in the MassIVE repository housed at the Center for Computational Mass Spectrometry at UCSD (http://proteomics.ucsd.edu/ProteoSAFe/datasets.jsp . The dataset has been assigned the MassIVE ID MSV000079442 and is available for FTP download at: ftp://MSV000079442@massive.ucsd.edu. The dataset will be locked until publication using the password “Candida”. The dataset was assigned the ProteomeXchange Consortium (http://proteomecentral.proteomexchange.org identifier PXD003421.

### Alkaline lysis of yeast cells (whole-cell extracts, WCE)

Yeast whole-cell extracts were prepared according to Khalimonchuk *et al. *79. Briefly, cells corresponding to A_600_ of 1.0 were pelleted by quick centrifugation (15 sec × 15,000 x*g*) and resuspended in 250 µl of 50 mM Tris-HCl, pH 8.0. Then, 50 µl of lysis buffer (10 M NaOH, 7.5% β-mercaptoethanol, 2 mM phenylmethylsulfonyl fluoride, PMSF) was added, and samples were vigorously mixed, followed by incubation on ice for 15 min. Then 220 µl of cold 100% TCA was mixed with the lysates, which were incubated on ice for a further 15 min. The samples were centrifuged at 10 min × 20,000 x*g* at 4°C. The pellets were washed twice with absolute acetone, dried on ice for 20 min, and resuspended in 133 µl of 1× Laemmli buffer followed by incubation at 85°C for 10 min.

### Isolation and purification of mitochondria

Mitochondria were isolated from 1 L cultures according to the previously described protocol 80 with slight modifications. The *2×FLAG-YDJ1/ydj1*Δ yeast strain was pre-grown in 50 ml of YPD for 16 hours. The pre-cultures were then split and used to re-grow in 1 L of YPD at 30°C and 37°C for 6 hours for further isolation and purification of mitochondria. Cells were collected by centrifugation for 5 minutes at 3,500 g at room temperature and then washed once with water. The wet weight of the pellets was then determined. Cells were subsequently resuspended in 0.1 M Tris-SO_4_, pH 9.4, 10 mM dithiothreitol (DTT) following the ratio of 0.5 g wet weight per 1 ml, and incubated at 30°C for 10 min. The cells were collected by centrifugation and washed once with 20 ml of 1.2 M sorbitol. The pellets were resuspended in 1.2 M Sorbitol, 20 mM KH_2_PO_4_, pH 7.4 with the ratio of 0.15 g wet weight per 1 ml. Lyticase (4 mg/g wet weight) was added to the cell suspension, and incubated at 30°C with gentle shaking (80 rpm) for 60 min. Obtained spheroplasts were collected by centrifugation and washed once in 30 ml of 1.2 M sorbitol. The pellets were resuspended in 10 ml of 0.65 M sorbitol, 10 mM Tris-HCl, pH 7.4, 1 mM PMSF and homogenized by 15 strokes in a tight-fitting Dounce homogenizer (40 ml, Kontes Glass Co.) on ice. The homogenates were centrifuged for 5 min at 3,500 x*g* at 4^o^C. The supernatants were collected into separate tubes and the pellets were re-homogenized a further two times. Combined supernatants were centrifuged for 10 min at 12,000 x*g* at 4^o^C. Supernatants were discarded, and accumulated lipids on the walls of the tubes were removed with paper towels. The remaining pellets were gently resuspended in 10 ml of 0.65 M sorbitol, 10 mM Tris-HCl, pH 7.4, 1 mM PMSF and centrifuged for 5 min at 2,500 x*g* at 4°C. Obtained supernatants were carefully transferred into new tubes and precipitated by centrifugation 10 min at 12,000g at 4°C. The supernatants were discarded and the pelleted mitochondria were resuspended in 750 ml of 0.65 M sorbitol, 10 mM Tris-HCl, pH 7.4, 1 mM PMSF.

Pure mitochondria were obtained by ultracentrifugation through a Histodenz (Sigma-Aldrich) gradient. The gradient was formed by adding 5 ml of 22% (w/v) Histodenz in 1.2M sorbitol and 10 ml of 14% (w/v) Histodenz in 1.2M sorbitol with a load of 1.5 ml crude mitochondria in 0.65 M sorbitol, 10 mM Tris-HCl, pH 7.4, 1 mM PMSF. Samples were centrifuged in a Beckman Coulter Optima( L-100K ultracentrifuge at 50,000 x*g* for 90 min at 4°C. The fractions of pure mitochondria were then collected from the interphase between 14% and 22% Histodenz, transferred into new tubes and filled up to 30 ml with 0.65 M sorbitol, 10 mM Tris-HCl, pH 7.4, 1 mM PMSF. The samples were precipitated by centrifugation for 10 min at 12,000 x*g* at 4°C. Obtained pellets were then resuspended in 1.5 ml of 0.65 M sorbitol, 10 mM Tris-HCl, pH 7.4, 1 mM PMSF and precipitated by centrifugation. Pure mitochondria were resuspended in 500 μl of 0.65 M sorbitol, 10 mM Tris-HCl, pH 7.4, 1 mM PMSF. Protein concentrations were measured by Bradford assay.

### Mitochondrial subfractionation

Mitochondrial protein topology was analyzed as described by Khalimonchuk *et al. *81. Six aliquots of isolated mitochondria (20 µg/sample) were resuspended in 1 ml of 0.65 M Sorbitol, 10 mM Tris-HCl, pH 7.4, and centrifuged for 10 min at 12,000 x*g* at 4°C. The pellets were resuspended in the following order: 1) intact mitochondria in 500 μl of 0.65 M Sorbitol, 10 mM Tris-HCl, pH 7.4; 2) intact mitochondria in 500 µl of 0.65 M Sorbitol, 10 mM Tris-HCl, pH 7.4 with 5 μl Proteinase K (100 µg/ml); 3) hypotonic swelling to selectively rupture the outer mitochondrial membrane in 500 μl of 20 mM HEPES, pH 7.4; 4) placed in 500 μl of 20 mM HEPES, pH 7.4 with 5 μl Proteinase K (100 μg/ml); 5) lysed mitochondria in 450 μl of 20 mM HEPES, pH 7.4 with 50 μl 10% n-dodecyl-β-D-maltoside (DDM); 6) placed in 450 μl of 20 mM HEPES, pH 7.4 with 50 μl 10% DDM and 5 μl Proteinase K (100 μg/ml). All samples were kept on ice for 30 min with gentle periodical mixing. After incubation 2.5 µl of PMSF (200 mM) was added to all samples. This was then followed by precipitation with 55 μl of 100% (w/v) trichloroacetic acid (TCA) added to every sample and vigorously mixed and treated as described above for whole-cell extract, except for adding DTT to a final concentration of 100 mM to 1× Laemmli buffer.

Carbonate extraction was performed as described by Fujiki *et al.* 82. Four aliquots of gradient-purified mitochondria (20 μg/sample) were centrifuged for 10 min at 12,000 x*g* at 4°C. The pellets were resuspended in 500 μl of the following solutions: 1) 0.65 M sorbitol, 10 mM Tris-HCl, pH 7.4, 1 mM PMSF; 2) 0.1 M NaHCO_3_, pH 8.25, 1 mM PMSF; 3) 0.1 M NaHCO_3_, pH 10.0, 1 mM PMSF; 4) 0.1 M NaHCO_3_, pH 11.5, 1 mM PMSF. The samples were incubated on ice for one hour followed by centrifugation at 60,000 x*g* for 45 min at 4°C. The pellets were resuspended in 40 μl of 1× Laemmli buffer, 100 mM DTT and incubated for 10 min at 85°C. The supernatants were transferred into new tubes and precipitated with 100% TCA as described above.

Sodium chloride extraction was performed similarly, however, pellets of four aliquots of gradient-purified mitochondria (20 μg/sample) were resuspended in total volume of 500 μl of 0.65 M sorbitol, 10 mM Tris-HCl, pH 7.4, 1 mM PMSF with the following final concentrations of NaCl: 0, 20, 100, and 500 mM.

Postmitochondrial fractions (PMFs) were obtained as following: the amount of cells corresponding to A_600_ of 2.0 were pelleted by quick centrifugation (15 sec × 15,000 x*g*) and resuspended in 500 μl of 0.65 M sorbitol, 10 mM Tris-HCl, pH 7.4, 1 mM PMSF. Approximately 300 μl of glass beads (0.5 mm) were added, and the samples were vigorously mixed for 5 min. The supernatants were transferred into new tubes. The beads were washed with 500 μl of 0.65 M sorbitol, 10 mM Tris-HCl, pH 7.4, 1 mM PMSF, and the collected supernatants were combined. The cell extracts were then centrifuged at 2 min x 4,000 x*g*. Obtained supernatants were carefully transferred into new tubes, and then centrifuged again at 20 min x 20,000 x*g*. Collected supernatants were then precipitated with TCA as described above.

### Co-immunoprecipitation assay

Mitochondrial protein interactions were assayed by coimmunoprecipitation as previously described 83. 300 μg of gradient-purified mitochondria were centrifuged at 12,000 x*g* at 4°C for 10 min. The pellets were resuspended in 50 μl of lysis buffer (1% (w/v) digitonin, 50 mM NaCl, 2 mM EDTA, pH 8.0, 1mM PMSF, 10 mM HEPES, pH 7.4) and lysed by gentle agitation at 4°C for 15 min. 1 ml of washing buffer (0.1% (w/v) digitonin, 50 mM NaCl, 2 mM EDTA, pH 8.0, 1mM PMSF, 10 mM HEPES, pH 7.4) was then added, and samples were centrifuged at 15 min x 16,000 x*g*, 4°C. 40 μl of the supernatants were mixed with 8 μl of 6× Laemmli buffer, incubated for 10 min at 85°C and used as a loading control for the Western blot. The rest of the supernatants (960 μl) were mixed with 40 μl of Myc-Tag (9B11) magnetic beads (Cell Signaling) and incubated for 24 hours at 4°C with gentle rotation. After incubation, the beads were precipitated by quick centrifugation at 15 sec x 15,000 x*g*, and a 40 μl aliquot of the supernatant was mixed with 8 μl of 6× Laemmli buffer, incubated for 10 min at 85^o^C and used as an unbound control for Western blotting. The beads were washed three times with washing buffer. Elution of coimmunoprecipitated fraction from the pelleted beads was performed in 40 μl of lysis buffer mixed with 8 μl of 6× Laemmli buffer at 72°C for 10 min with vigorous agitation. Samples were quickly centrifuged at 30 sec x 15,000 x*g*, and the supernatants were transferred to the new tubes to be used for Western blotting.
